# *Meloidogyne incognita PASSE-MURAILLE (MiPM)* Gene Encodes a Cell-Penetrating Protein That Interacts With the CSN5 Subunit of the COP9 Signalosome

**DOI:** 10.3389/fpls.2018.00904

**Published:** 2018-06-26

**Authors:** Caroline Bournaud, François-Xavier Gillet, André M. Murad, Emmanuel Bresso, Erika V. S. Albuquerque, Maria F. Grossi-de-Sá

**Affiliations:** ^1^Embrapa Genetic Resources and Biotechnology, Brasília, Brazil; ^2^Université de Lorraine, Centre National de la Recherche Scientifique, Inria, Laboratoire Lorrain de Recherche en Informatique et ses Applications, Nancy, France; ^3^Post-Graduation Program in Genomic Science and Biotechnology, Universidade Católica de Brasília, Brasília, Brazil

**Keywords:** interactome, root-knot nematode, affinity-purification, hub protein, cell entry, endocytosis, parasitism

## Abstract

The pathogenicity of phytonematodes relies on secreted virulence factors to rewire host cellular pathways for the benefits of the nematode. In the root-knot nematode (RKN) *Meloidogyne incognita*, thousands of predicted secreted proteins have been identified and are expected to interact with host proteins at different developmental stages of the parasite. Identifying the host targets will provide compelling evidence about the biological significance and molecular function of the predicted proteins. Here, we have focused on the hub protein CSN5, the fifth subunit of the pleiotropic and eukaryotic conserved COP9 signalosome (CSN), which is a regulatory component of the ubiquitin/proteasome system. We used affinity purification-mass spectrometry (AP-MS) to generate the interaction network of CSN5 in *M. incognita*-infected roots. We identified the complete CSN complex and other known CSN5 interaction partners in addition to unknown plant and *M. incognita* proteins. Among these, we described *M. incognita* PASSE-MURAILLE (MiPM), a small pioneer protein predicted to contain a secretory peptide that is up-regulated mostly in the J2 parasitic stage. We confirmed the CSN5-MiPM interaction, which occurs in the nucleus, by bimolecular fluorescence complementation (BiFC). Using MiPM as bait, a GST pull-down assay coupled with MS revealed some common protein partners between CSN5 and MiPM. We further showed by *in silico* and microscopic analyses that the recombinant purified MiPM protein enters the cells of Arabidopsis root tips in a non-infectious context. In further detail, the supercharged N-terminal tail of MiPM (NTT-MiPM) triggers an unknown host endocytosis pathway to penetrate the cell. The functional meaning of the CSN5-MiPM interaction in the *M. incognita* parasitism is discussed. Moreover, we propose that the cell-penetrating properties of some *M. incognita* secreted proteins might be a non-negligible mechanism for cell uptake, especially during the steps preceding the sedentary parasitic phase.

## Introduction

Root-knot nematodes (RKNs; i.e., *Meloidogyne* spp.) are considered the most ubiquitous and severe phytonematodes and can infect over 2,000 plant species, including many economically important crops (Trudgill and Blok, 2001; Decraemer and Hunt, 2013; Jones et al., 2013). Under favorable conditions, motile second-stage juveniles (pre-parasitic J2s) migrate and penetrate the elongation zone of the host root tip. Becoming infective J2s, the small animals intercellularly move up to reach the root vascular cylinder. After their stabilization in differentiated cells, the parasitic nematodes induce the formation of giant cells to serve as permanent nutrient sinks for the rest of the nematode's sedentary life cycle (Bartlem et al., 2014). The RKN infestation causes dramatic developmental changes via the formation of visible gall-like organs on host roots. At a physiological level, infective nematodes exploit key points of vulnerability in the host plant to subvert plant immunity and gain access to nutrients, thereby causing plant disease (Grundler and Hofmann, 2011; Bartlem et al., 2014; Toruño et al., 2016). Analyses of transcriptional profiles displayed obvious changes of host gene expression in response to the nematode infection (Barcala et al., 2010; Kyndt et al., 2012; Ji et al., 2013; Mendy et al., 2017; Yamaguchi et al., 2017). A comprehensive functional view of nematode effectors greatly contributes to deciphering their mode of action and to understanding how this small animal interferes with the host cellular machinery. Many nematode effectors manipulate different cellular pathways such as hormone signaling, protein post-translational modifications, redox signaling, cell wall modifications, or metabolism (Goverse and Smant, 2014; Hewezi, 2015; Holbein et al., 2016; Ali et al., 2017). While some of nematode effectors share similarities with known proteins, most of them are pioneer proteins making their characterization more challenging (Danchin et al., 2013; Rehman et al., 2016; Kikuchi et al., 2017).

Very little is known about the molecular mechanisms employed by secreted proteins to enter the host cell. Nematode effectors mainly originate from specialized secretory glands and are directly secreted through a protrusible stylet (Vieira et al., 2011; Mitchum et al., 2013). Others are also delivered along the nematode body, e.g., from the hypodermis onto the cuticle surface or from the chemosensory glands (called amphids) located on the head in the form of a prominent pore (Davis et al., 2008; Vieira et al., 2011). Ultrastructure studies of the plasma membrane of giant cells ingrowth revealed a minute hole that forms a direct connection between the apoplast and cytoplasm side through the stylet and the feeding tube respectively (Hussey and Mims, 1991; Mitchum et al., 2013). Some up-regulated effectors are early secreted by J2 pre-parasitic nematodes to neutralize the host oxidative pathway or suppress the programmed cell death by interacting with host proteins located inside the root cell (Rehman et al., 2009; Postma et al., 2012; Goverse and Smant, 2014; Ali et al., 2015; Holbein et al., 2016; Lin et al., 2016; Gillet et al., 2017; Habash et al., 2017). Moreover, secretome analyses of J2 pre-parasitic *M. incognita* exposed to root exudates have identified hundreds of proteins, of which a significant number are predicted to function inside the host cell (Bellafiore et al., 2008). This biological context underlined that certain nematode effectors are also delivered before the formation of the giant cells and the minute pore. Curiously, the immunocytochemical localization of such secreted effectors has been reported both in the apoplast or the cell wall and in the giant cell nuclei, where their function is expected (Lin et al., 2013; Chen et al., 2017). One question arises how these effectors reach their final destination in the host cell. An intuitive mechanism would be the secretion of such nematode proteins in the apoplast followed by entry into the host cell by an unknown mechanism. This hypothesis reminds some RxLR effectors in oomycetes that enter the host cell autonomously (Kale and Tyler, 2011). Remarkably, this feature is closely related to those described in cell-penetrating peptide (CPP), also called protein transduction domain (PTD) (Milletti, 2012). None “RxLR-like effectors” has been reported in phytonematodes so far (Hewezi and Baum, 2013; Eves-van den Akker et al., 2014). Nevertheless, a large variety of CPP/PTD families have been described in plants and animals (Chang et al., 2007; Cronican et al., 2011; Milletti, 2012; Liu et al., 2014; Chuah et al., 2015). In this regard, there is an intriguing possibility that an unexplored mechanism of host cell entry could exist for certain nematode effectors.

Although numerous nematode effectors have been identified, little is known about host proteins and biological processes that are preferentially targeted by the nematode. In general, protein networks are organized around a limited number of highly connected proteins, also called hubs. To borrow the metaphore of C. R. Landry, like a central station at an intersection in an urban subway network, hub proteins are located at the intersection of multiple cellular pathways involved in developmental and environmental responses (Yu et al., 2008; Dyer et al., 2010; Landry, 2011). In previous yeast-two hydrid studies, the interactome analysis of the Arabidopsis-pathogen immune network has highlighted that various effectors from bacterial, fungal, and oomycetic pathogens interact preferentially with 15 plant hubs (Mukhtar et al., 2011; Weßling et al., 2014). While these pathogens are separated by two billion years of evolutionary time, evolutionarily distant pathogens can convergently target common host cell signaling pathways. To our knowledge, no studies published thus far have focused on the identification of effector/hub protein interactions in phytonematodes. Certain “omics” analyses have reported that *M. incognita* secretes thousands of putative effector-like proteins during infection (Danchin et al., 2013; Nguyen et al., 2017). The identification of host targets of these nematode proteins may help to gain a better understanding of their putative biological function. As hubs undoubtedly contribute more than other proteins to shaping the host interactome, we postulated that some effectors, alone or in complex, could target these proteins to significantly facilitate RKN parasitism.

Among the hub candidates identified in Arabidopsis, the isoform CSN5a protein (also known as Jab1 or COPS5) is the most targeted host hub protein that interacts with at least 30 effectors secreted by various phytopathogens (Lozano-Duran et al., 2011; Mukhtar et al., 2011; Weßling et al., 2014). CSN5 is approximately 40 kDa protein and is highly conserved across kingdoms, which shares ~60% amino acid sequence identity between plants and animals (Barth et al., 2016). CSN5 is one of the eight subunits of the CSN complex, highly conserved amongst eukaryotes (Chamovitz and Segal, 2001; Barth et al., 2016). The CSN complex was first described in Arabidopsis as a photomorphogenic regulator of light-controlled plant growth and development (Wei et al., 1994; Chamovitz and Segal, 2001). The crystal structure of human CSN has been solved, providing a better understanding of the CSN complex as well as of CSN5 alone (Echalier et al., 2013; Lingaraju et al., 2014). CSN regulates the ubiquitin/proteasome system that specifically guides ubiquitylated proteins to the 26S proteasome for degradation (Schwechheimer, 2004). CSN interacts with numerous ubiquitin ligases, including the class of Cullin-RING E3 ubiquitin ligase complexes. The CSN role is the removal of the ubiquitin-like modifier NEDD8/RUB1, through the catalytic core of CSN5, to convert the ubiquitin ligase into an inactive form (Lyapina et al., 2001; Cope et al., 2002). The impairment of CSN alters the function of hundreds ubiquitin ligases involved in a wide variety of plant cellular processes such as hormonal pathways (e.g., auxin, jasmonate), the cell cycle, flowering time and immunity (Choi et al., 2014). Other CSN5 subcomplexes exist in the cytoplasm, but their roles and protein partners are still poorly defined (Wei et al., 2008; Schwechheimer and Isono, 2010). CSN5 interactome maps have revealed a large variety of interacting partners involved in the cell cycle, vesicle trafficking and immunity (Liu et al., 2009; Bennett et al., 2010; Mukhtar et al., 2011). Overall, CSN5 plays a central role in the coordinate regulation of cell machineries making it an attractive hub candidate to capture potential nematode effectors and to study of novel molecular mechanism in parasitism.

AP-MS approach allows the isolation and identification of proteins that interact with the “bait” protein of interest directly in a near-cellular environment (Chen et al., 2010; Morris et al., 2014; Van Leene et al., 2014; Dedecker et al., 2015). To address this challenge, we have developed a Strep-tag II purification system to isolate and identify interacting proteins of the CSN5a isoform within the pathosystem of interest *Nicotiana tabacum*—*M. incognita*. In addition, an attempt was to apply the engineered ascorbate peroxidase (APEX) to label nearby proteins of CSN5a in living cells. The labeled proteins are thereafter extracted, purified under harsh conditions, and identified by MS. The APEX system has emerged as a convenient and complementary research tool, which depicted interactome maps, for example, in human mitochondria (Rhee et al., 2013; Hung et al., 2014; Lam et al., 2015; Lee et al., 2016), in *Drosophila* (Chen et al., 2015), and more recently in yeast (Hwang and Espenshade, 2016), and *C. elegans* (Marx, 2015; Reinke et al., 2017). This approach has never been applied in a plant system, and unfortunately, our experimental APEX system failed in tobacco plants.

In this study, we generated transgenic plants to provide the first glimpse of protein-protein interactions toward CSN5a during *M. incognita* infection. The combination of proteomic and bioinformatic approaches led to the identification of Minc19205. We analyzed its expression profile during the nematode life cycle and identified some other plant protein partners in addition to CSN5a. The subcellular localization of the transiently expressed Minc19205 alone and when interacting with CSN5a was verified by fluorescence microscopy. The purification of recombinant Minc19205 combined with fluorescence microscopy enabled us to observe the cellular uptake of the protein in plant root tips. The protein's name, PASSE-MURAILLE (in English, “*The passer-through-walls*”), is related to its cell-penetrating properties and inspired by a fictional character from French literature (Aymé, 1947).

## Materials and methods

### Nematode and plant materials

Tobacco (*Nicotiana benthamiana* and *N. tabacum cv. “Petit Havana”*) and tomato (*Solanum lycopersicum* cv. “Santa Clara”) plants were grown in soil under greenhouse conditions with daily watering and subjected to natural light exposure. *M. incognita* race 1 was used for all experiments. The nematode culture and extraction were performed as described by (Atamian et al., 2012). Forty days after inoculation (dai), transgenic roots were collected and stored at −80°C until use.

### Molecular cloning of plasmids

The primers and nucleotide sequences of all constructs used in this study are given in Tables S3, S4. The soybean CSN5a-like ortholog was identified by BlastP search with the *A. thaliana* CSN5a protein sequence (accession number AT1G22920; Uniprot Q8LAZ7) as the query sequence by using the Phytozome website (version 10.0, phytozome.jgi.doe.gov). We identified the soybean CSN5a-like alias Glyma.04G075000.1 (Sequence ID KRH61926.1; Uniprot A0A0R0K5E1) by considering the score, similarity and identity percentages of hits with *E*-value < 1 e^−10^. The software Clustal X v2.1 was used to align CSN5 amino acid sequences from different species, including the Arabidopsis CSN5 isoforms and soybean CSN5a-like (Figure S1A). The phylogeny tree reconstruction has been built with the same CSN5 protein sequences using Phylogeny.fr (http://www.phylogeny.fr/). The APEX-tagged GmCSN5 construct (named APEX-GmCSN5 for convenience) was synthesized and cloned (Epoch Biolabs, USA, Houston) into the derived binary pGreenII vector (Hellens et al., 2000) to generate the plasmid pGIIH-azul-Strep-CSN5-APEX. The binary plasmid carried the blue fluorescent protein (BFP) for use as a positive reporter gene for plant transformation; it was constitutively expressed under the CaMV 35S promoter and specifically targeted to the mitochondria. With the aim to generate distinct plasmids expressing *MiPM*^−*SP*^ gene, we identified the *MiPM*^−*SP*^ gene sequence (sequence ID Minc19205) from the public database Wormbase Parasite (http://parasite.wormbase.org/index.html). The partial open reading frame (ORF) of *MiPM*^−*SP*^ was synthesized and cloned into the pBSK vector (Epoch Biolabs, Houston).

For subcellular localization and BiFC assays, *N. benthamiana* leaves were transiently transformed to express *GmCSN5* and *MiPM*^−*SP*^ constitutively in fusions with reporter genes. The *MiPM*^−*SP*^ ORF was digested and cloned with SalI and BamHI sites in frame into the full-length ORF encoding the enhanced green fluorescent protein (eGFP) (N- and C-terminal fusion) in the pEZS-NL and pEZS-CL plasmids (for subcellular localization assays). The *GmCSN5* and *MiPM*^−*SP*^ ORFs were digested and cloned with SalI and BamHI sites into the N- and C-terminus truncated ORF encoding a fragment of the yellow fluorescent protein (YFP) in the pEZS-NY and pEZS-YC plasmids (for BiFC assays).

To examine the role of MiPM^−SP^ in hypersensitive response (HR), the *MiPM*^−*SP*^ ORF was cloned into the pCAMBIA1300 vector with the NcoI and SacI restriction sites to generate pCAMBIA1300: MiPM^−SP^. The HR-elicitor INF1 of *Phytophthora infestans* was used as a positive control for HR (Bos et al., 2006). For more details about the HR assays in transgenic *N. benthamiana* leaves (Figure S4).

For pull-down assays, *MiPM*^−*SP*^ was cloned into the bacterial expression vector pETM-33b (Kanamycin resistance) to generate the plasmid pETM-33b:GST-MiPM^−SP^. The recombinant MiPM^−SP^ protein carried a 6xHis-tag at the N-terminal, followed by GST (glutathione S-transferase) and then the cleavage site HRV-3C and the MiPM^−SP^ sequence.

### Plant transformation

*N. tabacum* transformation was performed according to a previously described procedure (Clemente, 2006). T2 stable transgenic tobacco lines were screened under hygromycin, and the BFP fluorescence intensity in roots and leaves at different plant development stages was analyzed. A last round of selection was performed by the immunodetection of APEX-GmCSN5 with an antibody anti Strep-tag II. The representative tobacco line (named 11.11) has been selected and used in this study for APEX-GmCSN5 detection by western blot and for protein purification by affinity-based systems. The procedure for the APEX system from transgenic *N. tabacum* plants is described in the Figure S3.

### Subcellular localization and BiFC assays

Different plasmids of interest expressing the GmCSN5 and MiPM^−SP^ fusion proteins were bombarded onto detached and well-expended leaves of 3-week-old *N. benthamiana* plants by biolistic DNA delivery, as previously described (Morozov et al., 1997; Ueki et al., 2013). For BiFC assays, the following combinations of plasmids were tested: pEZS-NY-GmCSN5/YC-MiPM^−SP^ and pEZS-NY-MiPM^−SP^/YC-GmCSN5. A member of the basic Leu zipper (bZIP) transcription factor family in Arabidopsis, bZIP63 (AT5G28770), was used as a positive control by expressing the recombinant protein in the nucleus (Walter et al., 2004). Negative controls with plasmids expressing the NY or the YC fragment alone were bombarded in each BiFC experiment to verify the specificity of the interaction. At 24–48 h after bombardment, imaging of YFP fluorescence in the tobacco leaf epidermal cells was conducted using a confocal laser-scanning microscope (CLSM Leica SP8). The localisation of plant nuclei was confirmed by counterstaining with the blue fluorescing DAPI stain (4′,6- diamidino-2-phenylindole; 1 μg/ml) after incubation for 5 min in the dark. DAPI was excited at 405 nm (UV laser diode 405 nm) and detected within the emission wavelengths 420–490 nm. The reconstitued YFP was excited at 488 nm (laser OPSL 488) and detected within the emission wavelengths 515–550 nm. For the subcellular localization, the plasmids pEZS-NL-MiPM^−SP^ and pEZS-CL-MiPM^−SP^ were individually tested for subcellular localization in the epidermal cells of *N. benthamiana* leaves as described previously. The GFP was excited at 488 nm (laser OPSL 488) and detected within the emission wavelength 500–530 nm.

### Developmental expression analysis of MiPM in *M. incognita*

Total RNA was isolated from 100 mg of frozen and ground eggs, freshly hatched J2 nematodes, gall-enriched tissues from tobacco roots collected at 3 dai (parasitic J2) and 15 dpi (J3/J4) and fresh females collected at 25 dai. RNA was extracted by using TRIZOL, following the manufacturer's instructions (Invitrogen), and treated with DNAseI (1 U/μl, Invitrogen®). From each sample, 2 μg of DNase-treated RNA was used as a template for cDNA synthesis generated by using the MMLV Reverse Transcriptase (Invitrogen) and oligodT(30) (Macrogene). A single quantitative RT-PCR was performed in a 10 μl mixture including 1X of SYBR Green Master Mix, 2 μl of 1:10 cDNA, RNAse-free H_2_O and 0.2 μM of each forward and reverse primer (Table S3). PCR cycling parameters were set at 95°C for 15 min, followed by 40 cycles of 95°C for 15 s and 60°C for 1 min, with a melting curve step subjected to gradually increased temperature (from 60 to 94°C, reading every 0.5°C). The quantitative RT-PCR analyses were performed with a 7,500 Fast Real Time PCR system (Applied Biosystems). Each gene was analyzed in triplicate, and the results were generated from four independent biological experiments. Transcript levels were normalized against the geometric mean of two *M. incognita* housekeeping genes, *MiActin* and *Mi18S* (Arguel et al., 2012). Calculations were performed by the 2^−ΔCT^ method (Livak and Schmittgen, 2001).

### APEX-GmCSN5 protein expression

The protocol for protein extraction was adapted from a previous study (Van Leene et al., 2014) with some modifications. All steps described were performed at 4°C. Tobacco roots galls or leaves (0.5 g) were ground to powder in liquid nitrogen and homogenized in 1 ml of cold extraction buffer (EB) at pH 7.7 [100 mM Tris; 150 mM NaCl; 10 mM MgCl_2_; 0.1% CHAPS; 2.5 mM EDTA; 1 mM PMSF; 1 mM DTT; complete protease inhibitor cocktail (SIGMAFAST; Sigma^®;^) and 25 U/ml Benzonase (Promega) and 25 U/ml RNaseI (Invitrogen)]. The lysate was incubated for 1 h at 4°C on a tube rotator and subsequently centrifuged (14,000 rpm, 10 min, 4°C). The soluble fraction was separated on 12% SDS-PAGE and blotted onto nitrocellulose membrane (Amersham). Protran pore size 0.45 μm). The antibody Streptactin-AP (alkaline phosphatase) conjugate (Biorad) was used at 1:5,000 for the detection of GmCSN5 protein via the Strep-tag II. The colorimetric detection of AP activity was performed with p-nitro blue tetrazolium (NBT) as the substrate (Biorad).

### Purification of APEX-GmCSN5 protein complexes

The AP procedure involved the strong affinity of the Strep-tag II for the streptactin protein (a variant of streptavidin). The APEX-GmCSN5 protein partners were purified across three independent biological replicates. All of steps described were performed at 4°C in a cold-room. First, thirty grams of tobacco roots galls were manually ground in 100 ml of cold EB buffer during 30 min. The pH of the protein extract was controlled at 4°C. After centrifugation, the soluble fraction was filtrated (0.45 μm) and then applied to a gravity-flow chromatography column filled with 500 μl of equilibrated streptactin resin (GE Healthcare). After equilibration with 3CV (column volume) of MQ water (1 ml/min) followed by 5CV of lysis buffer (0.5 ml/min), the filtrate was continually loaded (1 ml/min). Unbound proteins were washed away with 10 CV lysis buffer, and then APEX-GmCSN5 and prey proteins were eluted with 5CV of lysis buffer supplemented with 10 mM D-desthiobiotin (Sigma). Throughout the process of protein extraction and purification, all fractions were collected and kept on ice until their separation on 12% SDS-PAGE gels. Protein bands were visualized by silver staining or Coomassie staining. The detection of APEX-GmCSN5 was confirmed by western blot as described above. After analyzing the protein fractions, samples were pooled and subsequently concentrated by acetone precipitation as previously described (Murad, 2011).

### Pull-down assays with GST-MiPM^−SP^

The capture of MiPM-interacting partners was achieved by GST pull-down assay as described previously (Michon et al., 2006) and performed from two independent biological experiments. First, 500 μg each of purified GST-MiPM^−SP^ and GST alone (as negative control) were immobilized on 500 μl GSTrap resin overnight in soft agitation at 4°C in PBS buffer. The resin was washed with 20 volumes of PBS buffer. Soluble proteins from *M. incognita*-infected tobacco root were incubated with the resin containing GST-MiPM^−SP^ and GST alone for 1 h. The resins were extensively washed and subsequently eluted with 30 mM reduced glutathione (50 mM Tris-HCl pH 8.0). All protein fractions were then separated on 12% SDS-Page with silver staining and immunoblotted with anti-Strep-tag II to detect APEX-GmCSN5 protein as described above. Protein samples were precipitated before LC-MS analysis according to a previous work (Murad, 2011).

### Mass spectrometry analysis and protein identification

The precipitated proteins were digested in-solution with trypsin and thereafter subjected to mass spectrometry analysis by methods previously described (Murad, 2011). Briefly, the tryptic fragments were separated using a 2D NanoUPLC system coupled into a SynaptG2 mass spectrometer (WATERS). NanoUPLC-MSe data were collected in an alternating low (3 eV) and elevated energy (ramp from 12 to 45 eV) mode of acquisition. The MS data was assessed by the Protein Lynx Global Server (PLGS) software (version 2.5.2) with Expression version 2 installed and conFigd to search proteins from the available protein sequence databases of *N. tabacum* (http://solgenomics.net) and *M. incognita* (http://www6.inra.fr/meloidogyne_incognita; INRA *Meloidogyne* resources). The criteria for the identification of proteins also included the detection of at least three fragment ions per peptide, six fragments per protein, the determination of at least one peptide per protein, and the identification of the protein was allowed with a maximum 4% false positive discovery rate. MS data were filtered by excluding any proteins identified by peptides in the control pull-down (GST alone) and in GST-MiPM^−SP^. The putative interacting proteins identified by affinity-based systems coupled with MS were next characterized by *in silico* analyses. To determine the identity of the prey proteins, peptide sequences were manually annotated by BlastP search from public databases to confirm their identity by using INRA *Meloidogyne* resources and Wormbase Parasite (http://parasite.wormbase.org/index.html) concerning nematode proteins and using National Center for Biotechnology Information (NCBI) for plants. The nematode protein candidates were subjected to a pipeline of available software (TargetP, TMHMM2.0, and Phobius) to predict the signal peptide and its respective cleavage site (Krogh et al., 2001; Emanuelsson et al., 2007; Kall et al., 2007). The physiological and chemical properties of *M. incognita* candidates were subjected to the Protparam tool of ExPaSy and the Genesilico Metaserver (http://web.expasy.org/protparam/; https://genesilico.pl/meta2) (Kurowski, 2003; Gasteiger et al., 2005).

### Labeling of MiPM^−SP^

The purified MiPM^−SP^ protein was labeled with the commercial fluorescent probe Alexa-488 from the Alexa Fluor protein labeling kit (Molecular Probes) according to the manufacturer's instructions. The protein was dialyzed (cut-off 3 kDa, 14–16 h, 4°C) in PBS buffer. The integrity and the fluorescence of the labeled protein (^*^MiPM^−SP^) were confirmed by SDS-Page with Coomassie blue staining and by gel-imaging equipment (Amersham Typhoon scanner imaging system) (ex. 488/em. 520).

### MiPM protein and NTT-MiPM uptake assays in arabidopsis roots

The putative NTT-MiPM was synthesized and labeled with the carboxyfluorescein probe (also named 5-FAM) at the N-terminal of its sequence by the Genescript Company. The experiment consisted of incubating 10 μM (0.1 mg/ml) of ^*^MiPM^−SP^ or 6xHis-eGFP and 1 μM of ^*^NTT-MiPM for 6 h in MG buffer (10 mM MgCl_2_ buffer pH 5.8) with Arabidopsis root tips. Before incubation, the non-conjugated Alexa488 probe (3 mM) was neutralized at 100 mM Tris-HCl (pH 8.5) to avoid unspecific labeling of root cells. The probe was next diluted at 1 μM in the MG buffer. Plants were washed three times 5 min in MG buffer, and the root tips were observed using a confocal laser-scanning microscope (CLSM Leica SP8). To improve the simultaneous detection of FM4-64 and the peptide in a short time scale, we increased the peptide concentration to 50 μM. Root tips were incubated for 30 min with FM4-64 and the peptide before a brief washing step with MG buffer. All images were acquired under identical microscopic parameters from two independent biological experiments (ex. 488/em. 520).

## Results

### *In silico* characterization of GmCSN5 and molecular strategies

The highly conserved CSN5 protein displays the canonical JAMM/MPN domain core, which is the catalytic center of CSN's deneddylase activity in eukaryotic organisms (Figure S1). In some plant species, CSN5 encodes two isoforms and assemble into distinct CSN complexes, but their functions still remains elusive (Gusmaroli et al., 2004; Jin et al., 2014). For instance, the Arabidopsis CSN5a and CSN5b isoforms share 86% amino acid sequence identity and exhibit a different role in plant development (Gusmaroli et al., 2004). The Arabidopsis CSN5a protein is currently known to be a privileged target for a wide range of effectors. We have thus identified the Arabidopsis ortholog AtCSN5a in soybean (alias Glyma.04G075000.1) with sequence identity and similarity above 80 and 86.6%, respectively from the publicly available database Phytozome (v.10). Given difficulties to generate soybean transgenic lines in our laboratory, we took advantage that CSN5a is widely conserved across multiple plant species (Figures S1A,B; Lozano-Duran et al., 2011) to use *Nicotiana tabacum* as valuable surrogate system for the generation of stable transgenic lines expressing the isoform CSN5a-like gene of soybean (named thereafter GmCSN5).

**Figure 1:**
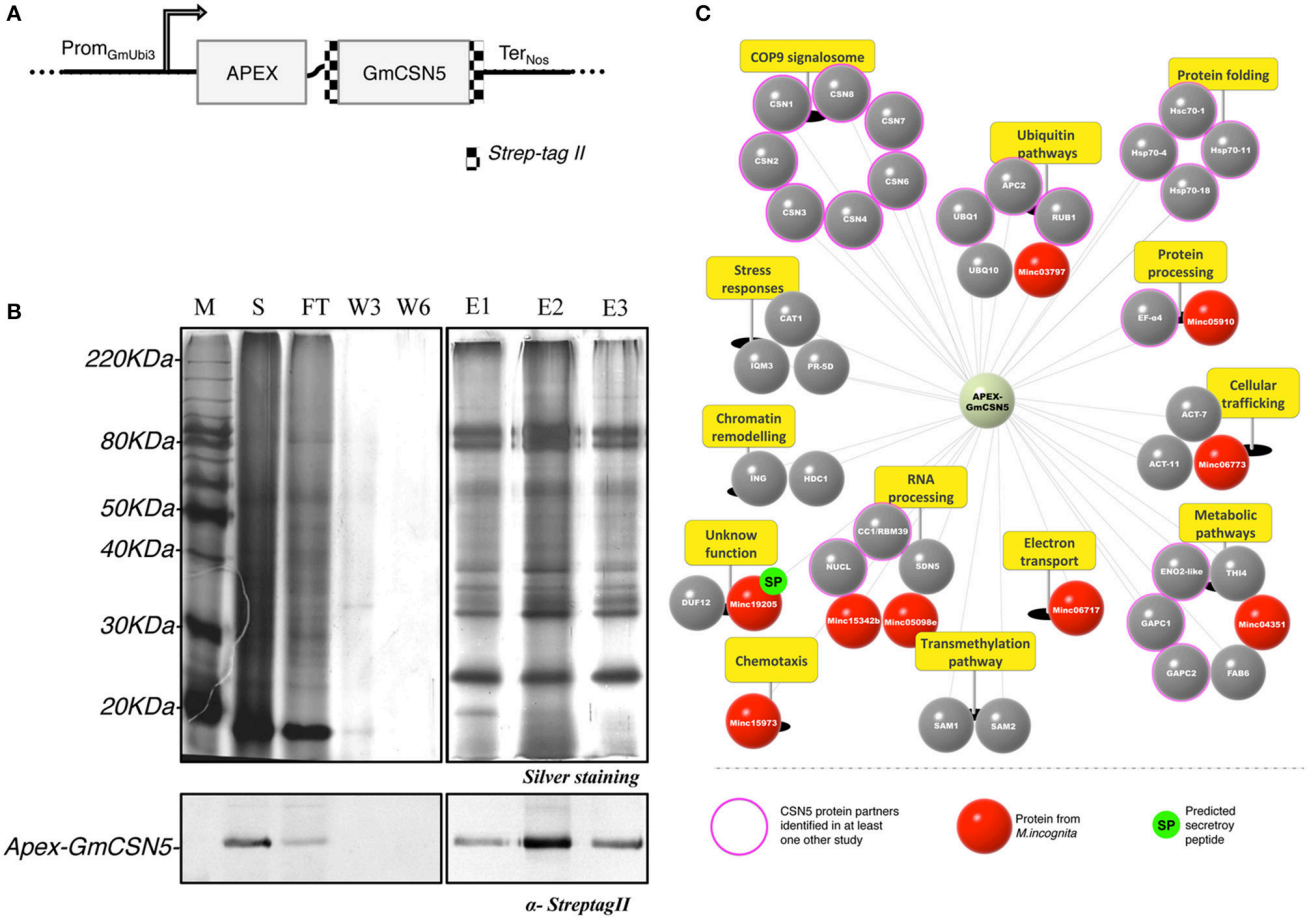
Root proteomic analysis of APEX-GmCSN5 protein partners during *M. incognita* infection. **(A)** Design of the construct APEX-GmCSN5 transformed into *N. tabacum*. The Strep-tag II is fused at the N and C-terminal parts of the GmCSN5 gene (alias Glyma.04G075000.1). A flexible linker was used to fuse APEX (~27 kDa) [Addgene number 49386; (Lam et al., 2015)] downstream to GmCSN5 (~40 kDa). The chimeric gene is expressed under a ubiquitin promoter (Prom_GmUbi3_; Hernandez-Garcia et al., 2010) and the nopaline synthase terminator (Ter_Nos_). **(B)** *M. incognita*-infected tobacco root extract of the line 11.11 was purified by StrepTrap chromatography with tagged APEX-GmCSN5 as bait. The protein samples for each purification step include the soluble fractions (S), flow-through (FT), washes (W3, W6), and eluates (E1, E2, E3) (top panel). Immunodetection of the Strep-tag II confirmed the presence of APEX-GmCSN5 in the soluble and elution fractions (~80 kDa) (bottom panel). **(C)** Snapshot of GmCSN5-interacting proteins identified by AP-MS and classified according to their biological processes (yellow panels). The scheme of the APEX-GmCSN5 interactome illustrates tobacco plant proteins in gray and nematode proteins in red. Conserved eukaryotic proteins known for their interaction with CSN5 in yeast and/or animal models are indicated by the outer pink circle. *M. incognita* proteins (named Minc) predicted to carry a putative secretory peptide are represented by a green circle labeled “SP.” AP-MS data was collected from three biological experiments.

Herein, we designed the construct to allow the capture of tagged-GmCSN5 protein complexes based on biochemical and proteomic strategies, respectively called StrepTrap-AP and APEX proximity-based labeling (Figure 1A). Given the solved crystal structure of the human CSN5 in the CSN complex (Echalier et al., 2013), we modeled the structure of the GmCSN5 in fusion with APEX and within the HsCSN complex (Figures S2A,B, Video S1). Docking simulation indicated low electrostatic interaction between the two proteins (Figure S2C), supporting a limited steric hindrance effect at the interface between GmCSN5 and APEX.

### Identification of candidate proteins interacting with APEX-GmCSN5

We first validated the molecular characterization of transgenic T2 *N. tabacum* lines (i.e., 11.7 and 11.11) stably expressing APEX-GmCSN5 by the evaluation of its protein expression in the roots before and after nematode infection (data not shown). In an attempt to identify the proteins in close proximity to GmCSN5, we examined the feasibility of the APEX system in plants. The methodology and the results obtained are described in the supplemental data section (Figure S3). In an effort to identify interacting partners of GmCSN5, the APEX system was unsuccessful.

We next assessed whether AP-MS was suitable at least to identify APEX-GmCSN5 in interaction with tobacco proteins related to CSN complexes. Proteins associated to APEX-GmCSN5 were purified by StrepTrap approach from transgenic tobacco roots infected with *M. incognita* (top panel, Figure 1B). The detection of APEX-GmCSN5 (~80 kDa) in the soluble and elution fractions was confirmed by western blot with the Strep-tag II antibody (bottom panel, Figure 1B). Enriched elution fractions were subjected to in-solution trypsin digestion, and the peptides were analyzed by nano-ultraperformance liquid chromatography nanoUPLC-MS^E^. A total of 43 proteins, including nine from *M. incognita*, were identified in three independent biological experiments (Figure 1C, Table S1). Candidate proteins identified by AP-MS were clustered according to their biological processes (Figure 1C).

As previously mentioned, CSN5 has been extensively described as a central regulator of the CSN complex (Chamovitz and Segal, 2001; Barth et al., 2016). It is worthy to note that we successfully identified all of eight tobacco CSN subunits (Figure 1C, Table S1). This result indicates that the heterologous expression of APEX-GmCSN5 can efficiently recruit the complete CSN complex of *N. tabacum*. Our outcome is also in agreement with our model predicting that the linker and APEX should not interfere with the CSN complex (Figure S2). In addition, we identified 20 proteins already known to interact with CSN5 protein (also known as Jab1 or COPS5), while the rest (almost 54%) were novel interacting proteins. The most highly represented group of proteins (~33%) belongs to the ubiquitin/proteasome system. Interestingly, orthologous proteins in Arabidopsis, such as CSN subunits and NEDD8/RUB1, bind either directly or indirectly with AtCSN5a (Arabidopsis Interactome Mapping Consortium, 2011). We also identified cullin-related protein APC2 (anaphase promoting complex subunit 2), which is known to be involved in the formation of polynucleated cells during *M. incognita* parasitism (de Almeida Engler et al., 2012). Another large group (~12%) includes the HSP (heat-shock protein) and HSC (heat-shock cognate) protein families, which are mostly related to stress responses and involved in protein trafficking, degradation, or immunity (Park and Seo, 2015; Schorey et al., 2015; Fernández-Fernández et al., 2017). While the roles of these protein-protein interactions remain unclear (Bennett et al., 2010), another study has proved that CSN5 contributes to the regulation of protein sorting into exosomes, such as HSP70 proteins, in a ubiquitin-dependent manner (Liu et al., 2009). Consistent with the function of CSN5 in the nucleus, other co-purified proteins involved in the RNA processing pathway were detected, e.g., the splicing factor CC1/RBM39, also called CAPERα, which is known to be a protein partner of CSN5 in animal (Bennett et al., 2010). CC1/RBM39 is widely conserved among eukaryotes, with 43.1% amino acid sequence identity shared between *A. thaliana* and humans. In addition, other proteins appear to play a role in cellular trafficking, such as actins that contribute to mediating cellular movement (e.g., membrane recycling, carrier formation, cargo sorting). We also noted the occurrence of proteins involved in primary metabolism (~22%), stress responses (~9%), and chromatin remodeling (~6%).

We have also reported the capture of nine proteins from *M. incognita*, five of which are clustered in similar biological processes to those found for the co-purified tobacco proteins. Two nematode proteins (Minc05098 and Minc15342) involved in RNA processing are classified as U2 small nuclear ribonucleoprotein splicing factors (U2snRNPs). These nematode proteins show similarities with the previously mentioned tobacco CC1/RBM39 protein. One harbors a zinc finger RING-type domain (Minc03797) and could be a new type of RING E3 ligase. We also identified an actin protein (Minc06773), another conserved protein related to the trafficking pathway. Four other nematode proteins have not yet been described but show similarities to the following: cytochrome c/b562 protein (Minc06717), *C. elegans* nas-5 protein (Minc05910), and *C. elegans* Ly-6-related HOT-3 (Minc15973). The last, Minc19205 (named *M. incognita* PASSE-MURAILLE, MiPM), is a unique protein in *M. incognita* without clear similarities to other known proteins, for which gene expression has already been reported during parasitism (Nguyen et al., 2017).

Altogether, the identification of the entire CSN complex and other known partners from the ubiquitin/proteasome system highlights the reliability of our methodology. The identification of new potential interacting proteins, sometimes in agreement with the predicted CSN5 functions, might contribute to a better understanding of the role of this hub in plant-pathogen interactions. Accordingly, our assay does not exclude that some candidate proteins interact with APEX.

### MiPM is expressed early during nematode parasitism

**Figure 2:**
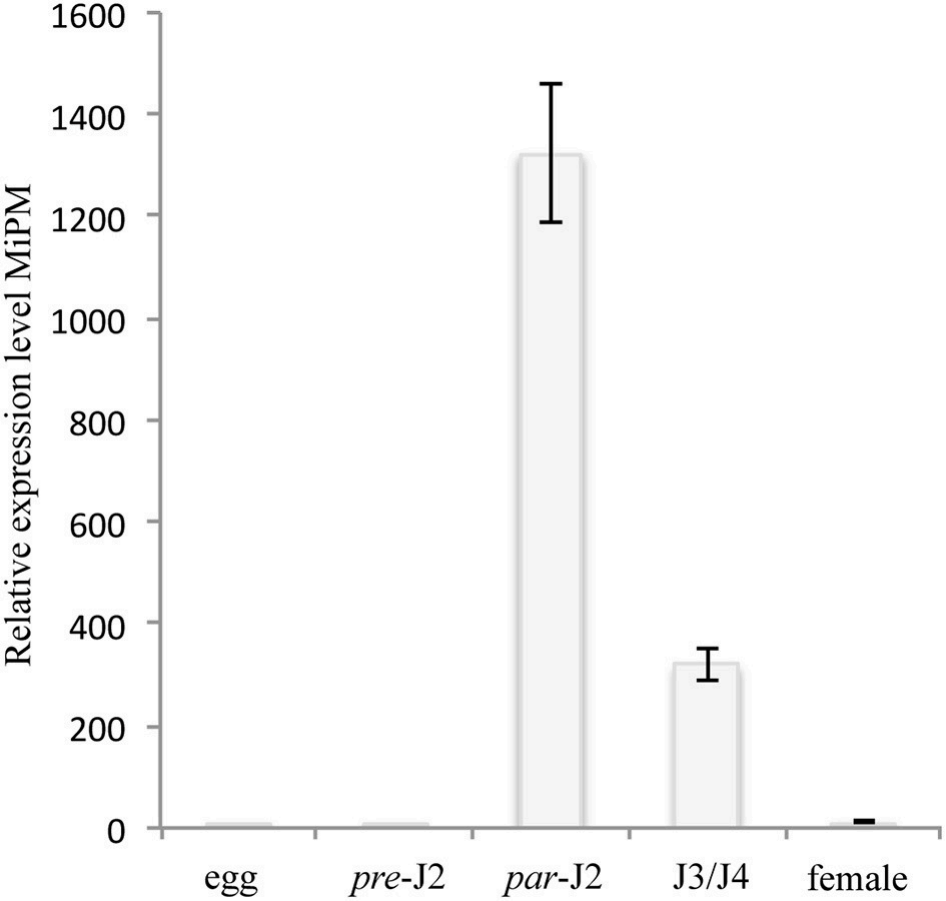
MiPM expression is highly up-regulated during the parasitic J2 stage of *M. incognita*. The MiPM expression level was evaluated by quantitative RT-PCR at different stages of development of *M. incognita*, i.e., eggs, pre-infective second juveniles (pre-J2), parasitic second juveniles (par-J2; 3 dai), third- and fourth-stage juveniles (J3/J4; 15 dai) and females (25 dai). Fold change values were calculated by the 2^−(−ΔΔ*Ct*)^ method, and the data were normalized according to the geometric average of two reference genes (*MiActin* and *Mi18S*) and relative to the expression in eggs (Livak and Schmittgen, 2001; Arguel et al., 2012). Each column represents the mean and standard deviation of four independent biological experiments.

One of the important typical characteristics of nematode effector proteins is the presence of a N-terminal secretory peptide (SP) (Danchin et al., 2013; Nguyen et al., 2017). Only MiPM was predicted with high likelihood to contain a secretory peptide (cut-off 0.9). The occurrence of the MiPM gene in the *M. incognita* genome was verified by PCR amplification, and the flanking regions were sequenced. We next investigated whether MiPM is expressed during nematode parasitism. The transcriptional expression of MiPM was assessed in different developmental stages of *M. incognita* by using quantitative RT-PCR. Compared to the egg stage, MiPM is transcriptionally highly up-regulated at early time points during the nematode infection. The expression of MiPM was significantly higher in parasitic J2s (3 dai) and, then there was a gradual decreasing in J3/J4 (15 dai) and in female stages (25 dai) (Figure 2). This result suggests that MiPM acts during early events in nematode infection, since infective nematodes penetrate and migrate within host root tips.

We next examined whether MiPM plays a possible role in plant immunity by activating or suppressing the hypersensitive reaction (HR) (Figure S4). In *N. benthamiana* leaves infiltrated with *A. tumefaciens* expressing MiPM without its secretory peptide (MiPM^−SP^), we did not observe HR at any time, in contrast to those infiltrated with the oomycete HR-elicitor INF1 (used as a positive control) (Bos et al., 2006). In addition, the ability of MiPM^−SP^ to suppress INF1-triggered cell death was not observed in *N. benthamiana* leaves under our experimental conditions. Taken together, these results underline that the function of MiPM in plant immunity remains inconclusive.

### GmCSN5 interacts with the nematode MiPM protein

**Figure 3:**
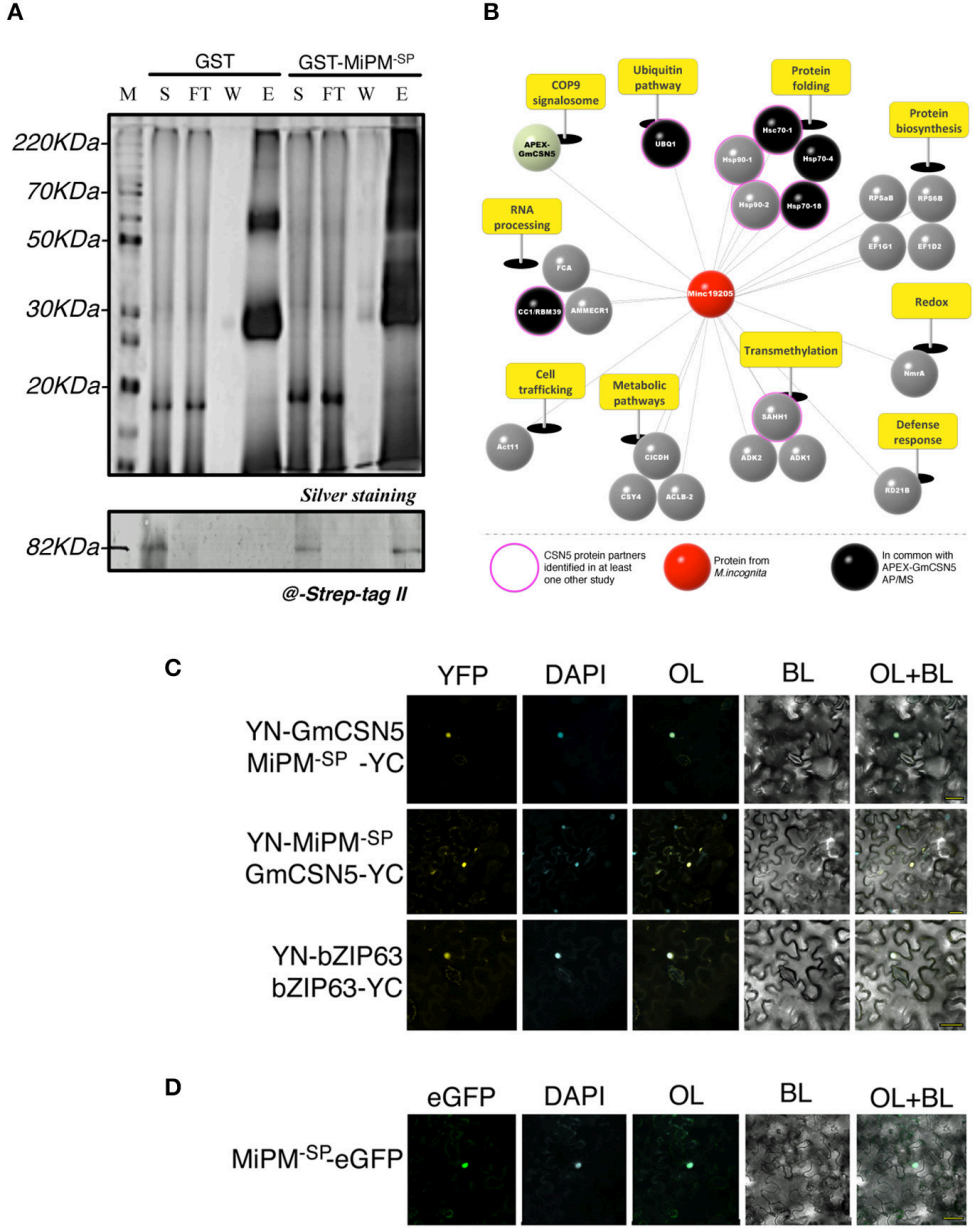
MiPM and GmCSN5 interact together in the nucleus and share common protein partners. **(A)** Pull-down assay of soluble proteins from *M. incognita*-infected tobacco roots of the line 11.11 with the recombinant proteins GST-MiPM^−SP^ and GST (as negative control). The soluble fraction (S), flow-through (FT), washes (W), and eluates (E1) were separated by SDS-PAGE (top panel) and analyzed by silver staining. GST- MiPM^−SP^ and GST migrate around 38 and 28 kDa, respectively. APEX-GmCSN5 detection (~80 kDa) was performed by western blot using anti-Strep-tag II antibody (bottom panel). **(B)** Snapshot of MiPM^−SP^ interacting proteins identified by GST pull-down assay followed by MS. The identified proteins were classified and labeled as mentioned in Figure 1C. Proteins identified in association with either APEX-GmCSN5 or MiPM^−SP^ are indicated in black. MS data was collected from two independent biological experiments. **(C)** The protein interaction of MiPM^−SP^ with GmCSN5 (devoid of APEX) was confirmed by *in planta* BiFC assays. At 24–48 h after co-bombardment with the combination YN-GmCSN5/MiPM^−SP^-YC and YN-MiPM^−SP^/GmCSN5-YC, the reconstituted YFP fluorescence signal is observed in the bombarded *N. benthamiana* leaf epidermal cells. The co-localisation of YFP and DAPI fluorescence indicates that MiPM^−SP^ interacts with GmCSN5 in the nuclei. BZIP63 is the positive control, as its dimerization occurs in the nucleus. **(D)** Ectopically expressed MiPM^−SP^-GFP co-localizes to the cell nucleus with the DAPI stain in *N. benthamiana* leaves. All images were taken 24–48 h after bombardment across two independent biological experiments. BL, bright light; OL, overlay.

We next verified the interaction between GmCSN5 and MiPM. We first performed a GST pull-down assay using the recombinant protein GST-MiPM^−SP^ or GST alone (as negative control) with the whole lysate of *M. incognita*-infected root extracts overexpressing APEX-GmCSN5. The production and purification of the recombinant proteins are presented in the Figure S5. Eluted proteins associated with GST-MiPM^−SP^ or GST were separated and visualized by silver-stained SDS-PAGE (top panel; Figure 3A). GST-MiPM^−SP^-bound resin, but not the GST control, provided enrichment in APEX-GmCSN5 detected by western blot with the strep-tag II antibody (bottom panel; Figure 3A). These data suggest that MiPM is associated with APEX-GmCSN5 protein complexes.

To further identify host proteins that interact with MiPM, we subjected proteins co-purified with GST-MiPM^−SP^ to NanoUPLC-MSe analysis from two independent biological repetitions (Figure 3B; Table S2). Six proteins were previously found by AP-MS and are mainly known as partners of CSN5: three molecular chaperones (HSP70-18, HSC70-1, HSC70-4), a ubiquitin protein (UBQ1), a splicing factor (CC1/RBM39), and the CSN subunit (CSN5). We identified 23 host proteins, but none from *M. incognita*. Identified proteins are classified according to 10 biological processes including protein folding (~22%), RNA processing (~13%), and ubiquitination (~4%). Furthermore, we identified one protein ortholog of Arabidopsis papain-like cysteine proteases (PLCPs), which is named RD21B protein and is considered a hub protein in plant-pathogen interactions (Misas-Villamil et al., 2016). Altogether, these observations are consistent with the previous AP-MS experiments and support the hypothesis that MiPM^−SP^ can interact with APEX-GmCSN5.

While AP and GST pull-down experiments have shown that MiPM interacts with APEX-GmCSN5, our results do not rule out that MiPM specifically binds to GmCSN5 rather than APEX. Thus, we further tested the interaction GmCSN5 (devoid of APEX)—MiPM^−SP^ *in planta* by using bimolecular fluorescence complementation (BiFC). The coding sequences of MiPM^−SP^ and the full-length GmCSN5 were fused N-terminally to the coding sequences of the non-fluorescent halves of yellow fluorescent protein (YFP) and co-bombarded in *N. benthamiana* leaves. The Arabidopsis transcription factor bZIP63, known to homodimerize in the nucleus, was used as a positive control (Walter et al., 2004). As expected, bZIP63 co-expression led to the detection of YFP fluorescence co-localized with the DAPI nuclear marker. Similar to the positive control, the co-localization of YFP and DAPI indicates the interaction of GmCSN5 with MiPM in the nucleus (Figure 3C). By contrast, co-expression of any those constructs with the empty YN or YC constructs did not reconstitute the YFP fluorescence (Figure S6). We next investigated whether MiPM^−SP^ occurs in other subcellular compartments. Two constructs were generated by fusion MiPM^−SP^ to the N- or C-terminal part of the full-length GFP. We observed MiPM^−SP^-GFP mostly in the nuclei and did not observe any signals for the construct GFP-MiPM^−SP^ (Figure 3D; Figure S7). In sum, the GST pull-down assay also reveals the interaction of APEX-GmCSN5 as shown by AP-MS, and the BiFC analysis shows that MiPM^−SP^ interacts with GmCSN5 in the nucleus.

### Functional characterization of MiPM as potential cell-penetrating protein

**Figure 4:**
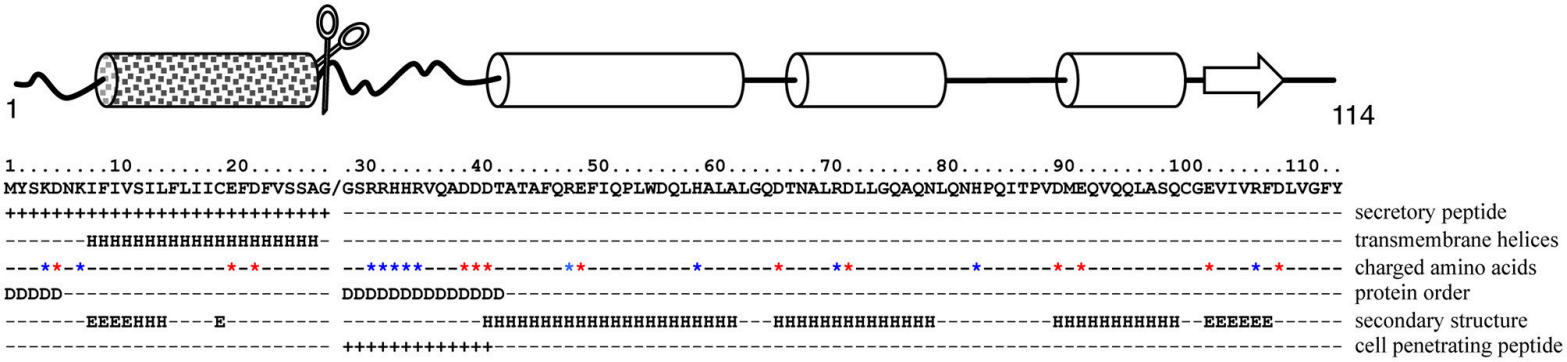
*In silico* analysis suggests that MiPM is mostly a folded protein with a putative CPP/PTD in its N-terminal tail. In the scheme, arrows and cylinders depict the putative secondary structures, ß-sheets and α-helices respectively. The square-motif cylinder represents the predicted transmembrane helix containing the secretory peptide (SP). The chisel-shaped section is the predicted SP cleavage site. In the text box, the location of the predicted SP and CPP is indicated by “+.” Aspartate (E) and glutamate (D) are indicated by a red star and arginine (R), lysine (K) and histidine (H) by blue stars. “D” indicates the prediction of a disordered region, while secondary structures are labeled “H” for helices and “E” for beta strands.

To provide putative mechanistic details, we then performed an *in silico* analysis of MiPM (Figure 4). As previously indicated, a secretory peptide comprising a transmembrane domain was predicted in the amino acid region 1–26 (cut-off 0.9). The protein is acidic (pI 5.03) and small, with a molecular weight of 10 kDa without the predicted secretory peptide. By using 17 prediction models, the consensus secondary structure prediction reported a robust model in which ~72% of the sequence might be folded into alpha helix structures. Thus, MiPM is predicted to be a stable protein. The region 27–39, located downstream of the secretory peptide, drew particular attention. We hypothesized that this region presents properties similar to CPP/PTDs. This region is supercharged and an intrinsic disorder is predicted. Given this configuration, the residues might be solvent-exposed. Moreover, the prediction of the secretory peptide suggests that MiPM might be released in the apoplast of the plant root at the J2 parasitic stage during the intercellular migration. Notably, the pH of the extracellular space is usually estimated to be ~5–6 in plants (Geilfus, 2017). Histidine (H), with a pKa of ~6.5, tends to be protonated in the extracellular space. Given these conditions, we thus supposed that dual histidine (H) and arginine (R) (pKa ~12.4) residues generate high positive charges in this region. Finally, the N-terminal tail “GSRRHHRVQADDD” was predicted as a putative transduction domain in MiPM (confidence 0.730). Altogether, our *in silico* analysis suggests that MiPM is a unique putative secreted protein, probably well folded and containing a putative CPP/PTD region.

**Figure 5:**
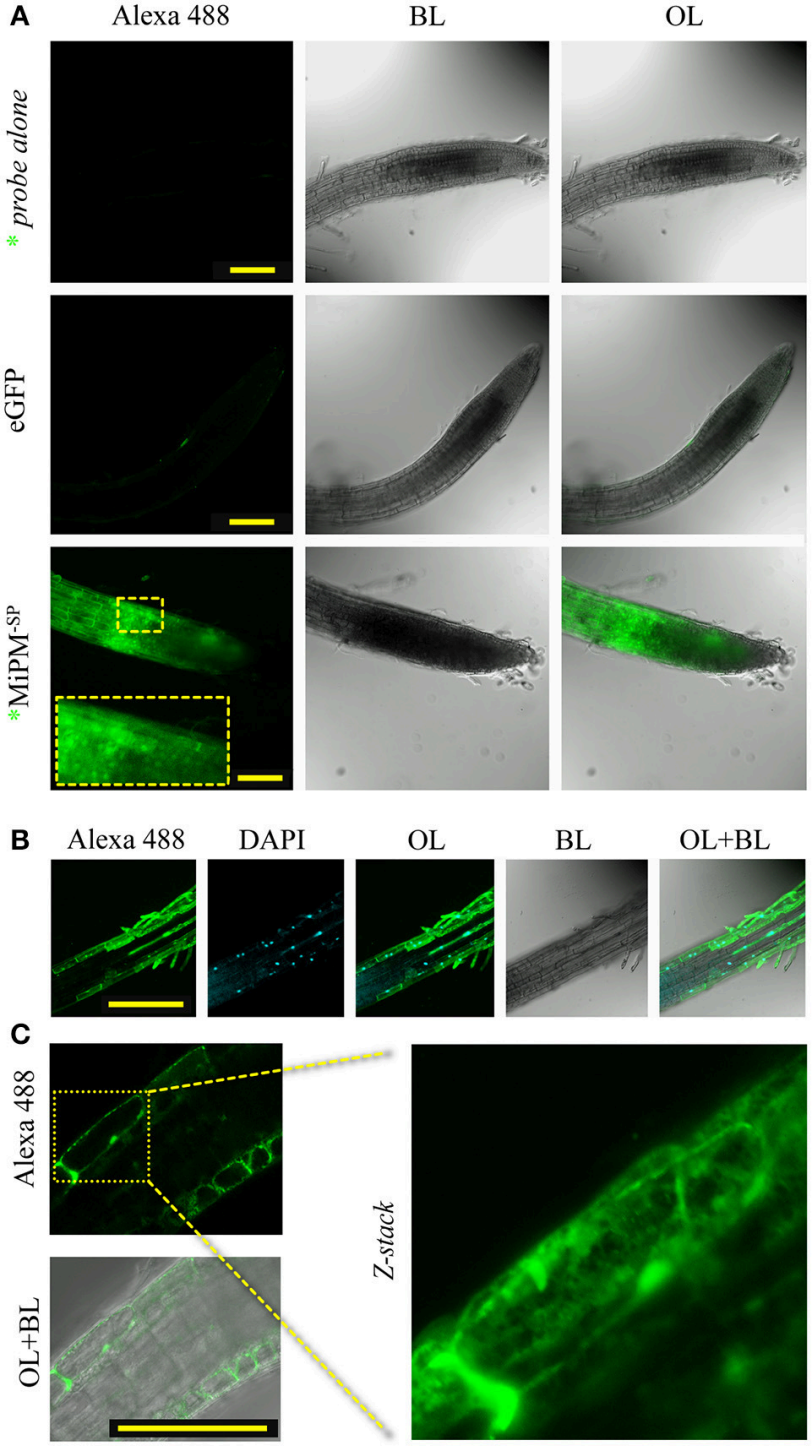
MiPM^−SP^ is internalized in Arabidopsis root cells. The purified recombinant protein MiPM^−SP^ was labeled with Alexa488 (*MiPM^−SP^) (Figure S9). Root tips of Arabidopsis seedlings were incubated with 10 μM (0.1 mg/ml) of *MiPM^−SP^, 6xHis-eGFP or inactivated Alexa488 probe alone (as a negative control) for approximately 6 h in the dark before observation by confocal microscopy. **(A)** A general overview of the Arabidopsis root tips reveals the accumulation of the green fluorescence of *MiPM^−SP^ within plant cells. **(B)** *MiPM^−SP^ co-localizes with DAPI inside the nucleus. **(C)** *MiPM^−SP^ fluorescence shows numerous punctate spots and extended spans within the root cell. BL, bright light; OL, overlay; λ488–520, spectral range for eGFP and Alexa488 detection in nanometers. Bars, 100 μm.

To assess our hypothesis, we investigated by fluorescence microscopy whether MiPM could translocate across the plasma membrane in plant cells. The cellular uptake assay is widely used for characterizing protein translocation in animals (Kale et al., 2010; Cronican et al., 2011; Boddey et al., 2016) and plants (Chang et al., 2007; Dou et al., 2008; Kale et al., 2010; Plett et al., 2011; Eggenberger et al., 2016). However, the methodology of the protein uptake assay is currently under debate (Tyler et al., 2013; Wawra et al., 2013; Petre and Kamoun, 2014). Considering this controversy, we wished to take additional precautions to avoid spurious interpretations. Indeed, the use of tandem repeat histidines, like 6xHis-tag, has been found to slightly promote cell entry in human cells (Liu and Gao, 2013). We previously reported the production of 6xHis-GST-MiPM^−SP^ (Figure S5). A HRV-3C proteolytic cleavage site was inserted between 6xHis-GST and MiPM^−SP^ to cause the separation of the two proteins. Thus, the MiPM^−SP^ used in our bioassays is His-tag free. Subsequently, the recombinant protein was isolated by gel filtration (Figure S8). To investigate the translocation mechanism of MiPM, we labeled the purified MiPM^−SP^ at its N-terminal with Alexa488 fluorescent probe (named ^*^MiPM^−SP^). The protein was freshly purified, and its integrity after the purification process was verified by SDS-PAGE followed by fluorescence imaging (Figure S9). While no significant fluorescent signal was detected with the neutralized Alexa488 probe alone or with 6xHis-eGFP, a strong intracellular accumulation of ^*^MiPM^−SP^ was visible in the root cells (Figure 5A). Notably, a fluorescent signal becomes clear when the contrast and lightness of the picture is increased and shows slight translocation of 6xHis-eGFP. Probably due to the absorption function of hairy root cells, we also detected a strong Alexa488 probe signal in the hairy root cells (data not shown). ^*^MiPM^−SP^ co-localized with DAPI, indicating that the protein accumulates in the nuclei (Figure 5B). In greater detail, ^*^MiPM^−SP^ could be observed in a network spanning the cell and in vesicle-like bodies inside the cell (Figure 5C). Overall, this experiment demonstrates that ^*^MiPM^−SP^ translocates into plant root tip cells and localizes in different subcellular compartments.

**Figure 6:**
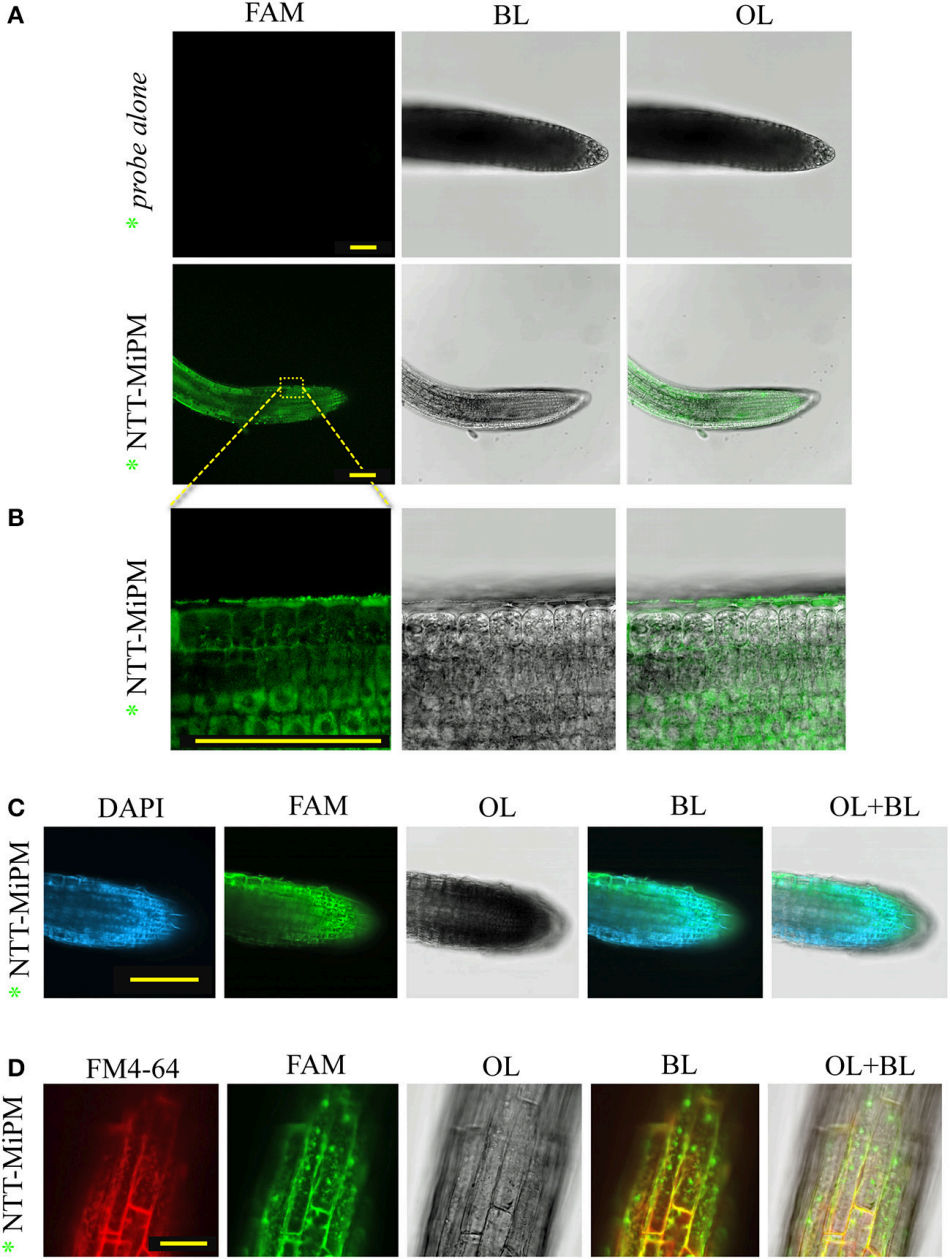
NTT-MiPM has cell-penetrating properties in Arabidopsis root cells. The root tips of Arabidopsis seedlings were incubated with 1 μM of synthetized NTT-MiPM conjugated with 5-FAM (*NTT-MiPM) for ~4 h in the dark before observation by confocal microscopy. **(A)** A general overview of the Arabidopsis root tips shows the accumulation of green fluorescence corresponding to *NTT-MiPM within plant cells. **(B)** *NTT-MiPM localizes in vesicle-like bodies but not in the nucleus. **(C)** *NTT-MiPM does not co-localize in the nucleus with DAPI. The root tips of Arabidopsis seedlings were incubated with the DAPI stain 1 h before adding *NTT-MiPM (50 μM) for ~30 min in the dark and then observed by fluorescence imaging. **(D)** Co-localization of the membrane marker FM4-64 indicates that *NTT-MiPM uses an endocytic pathway to penetrate the host cell. BL, bright light; OL, overlay; λ488–520, spectral range for eGFP and Alexa488 detection in nanometers. Bars, 100 μm **(A–C)** and 30 μm **(D)**.

We next examined whether the N-terminal tail of MiPM (i.e., “GSRRHHRVQADDD,” named NTT-MiPM) can autonomously trigger cell uptake. The synthetized peptide was conjugated with 5-FAM fluorescent marker at the N-terminal (annotated ^*^NTT-MiPM). Arabidopsis root tips were incubated with the peptide (1 μM) or with the 5-FAM probe alone (Figure 6). The peptide ^*^NTT-MiPM, but not the probe alone, resulted in the detection of a fluorescent signal in the root cells (Figures 6A,B). However, no co-localization into the nuclei was detected between ^*^NTT-MiPM and the DAPI stain (Figure 6C). Rather, it appears that the peptide accumulates in vesicle-bodies or in the vacuole. It is often reported that CPP/PTDs trigger the endocytosis pathway to translocate across the plasma membrane (Madani et al., 2011). Therefore, we postulated that the punctate pattern might correspond to vesicle-like bodies. To assess this hypothesis, Arabidopsis seedling roots were co-incubated with ^*^NTT-MiPM and the fluorescent endocytosis marker FM4-64 (Figure 6D). Notably, FM4-64 penetrates the cell within minutes and can provoke high background fluorescent signals. To improve the simultaneous detection of FM4-64 and the peptide in a short time scale, we increased the peptide concentration to 50 μM. Root tips were incubated for 30 min with FM4-64 and the peptide before a brief washing step. The resulting punctate fluorescence signals formed clusters and partially co-localized with the labeled endovesicles, which were marked by both FM4-64 and the fluorescent peptide. Altogether, these results indicate that NTT-MiPM can translocate across the plasma cell membrane of the root tip cells. We also show that the peptide stimulates an unidentified endocytosis pathway.

## Discussion

### Isolation and identification of nematode proteins interacting within GmCSN5 protein complexes

Tremendous efforts in “omic” technologies have identified a variety of nematode effectors. The next challenge is to understand how they act together to modulate the host interactome for the benefit of the pathogen. GmCSN5 was an interest for characterizing the interacting pathogen components from *M. incognita* via biochemical and proteomic approaches. In this study, we originally expected AP-MS to be a complementary approach to APEX system. Owing to a lack of conclusive data, the AP-MS approach was finally adopted as an alternative. Herein, we have successfully identified GmCSN5 protein partners under near physiological conditions and in nematode infection. The identification of the complete CSN complex with additional known partners supports the robustness of the AP-MS methodology. Overall, we experimentally identified 34 proteins, including nine *M. incognita* proteins. The capture of *N. tabacum* proteins with the soybean GmCSN5 supports the fact that these interactions are evolutionarily conserved (Bennett et al., 2010; Lozano-Duran et al., 2011; Barth et al., 2016). Notably, the discovery of nematode proteins associated with these complexes in a heterologous context allowed the illumination of some common signaling pathways targeted by the nematode in a variety of wild hosts. Unexpectedly, few proteins were identified in our AP-MS approach, which is particularly surprising given that CSN5 is a hub protein. For comparison, a previous study reported the identification by AP-MS of 640 proteins interacting with CSN5 in human cells (Bennett et al., 2010). Considering the biological system employed, the accurate protein complex retrieval is particularly sensitive (Dedecker et al., 2015). In our study, transgenic plants grew directly in the soil, thus even after cleaning; some traces of soil might interfere with the stability of protein complexes during protein extraction. Overall, we gained a broader view of the potential pathogen components that target GmCSN5 under microbial infection.

### MiPM is a pioneer protein expressed during nematode infection

MiPM is a pioneer gene encoding a protein without similarities to known proteins and containing no detectable functional domains. Nevertheless, it shares at least one common trait with many of the potential secreted proteins in *M. incognita* (Huang et al., 2003; Danchin et al., 2013; Nguyen et al., 2017). Previous studies suggest that *M. incognita* pioneer genes encoding effectors act as potential host specificity determinants (Jaouannet et al., 2012; Rutter et al., 2014; Xie et al., 2016; Nguyen et al., 2017). Herein, transcriptional expression analysis reveals that MiPM is highly expressed at the parasitic J2 stage, when the nematode secretes massive amounts of effectors and promotes the formation of the giant cells (Sijmons et al., 1994; Hewezi, 2015; Rehman et al., 2016). Our data indicates that the putative secreted MiPM protein acts during nematode infection but without triggering HR. Moreover, our experimental assay did not integrate all of components and complexity of an infectious context. The methodology here implied MiPM^−SP^ transiently expressed in the cells of *N. benthamiana* independently of an infectious context. The deciphering of the *Burkholderia*-human protein interaction network has revealed that multiple pathogen proteins can target the same host protein (Memišević et al., 2015). Similar features have been reported for virulence effectors in various Arabidopsis-pathogen interactomes (Mukhtar et al., 2011; Weßling et al., 2014). MiPM could likewise function in complex with other Minc proteins to effectively interfere with HR or others pathways in plant immunity. Remarkably, MiPM^−SP^ pulled-down with a putative secreted PLCP called NtRD21B. PLCPs induce defense responses and cell death and are also commonly targeted by pathogen effectors from fungi (Shindo et al., 2012; Lampl et al., 2013), oomycetes (Kaschani et al., 2010; Bozkurt et al., 2011), and phytonematodes (Lozano-Torres et al., 2012, 2014; for review Misas-Villamil et al., 2016). The MiPM^−SP^ role as potent activator of plant immunity in the apoplast might be investigated. Some deeper molecular explorations on the interaction of MiPM and plant immunity are necessary to decipher the function of MiPM in parasitism.

### MiPM might enter the plant cell via endocytosis

MiPM has cell-penetrating properties, as demonstrated by protein uptake experiments in non-infected Arabidopsis roots. Our comprehensive *in silico* and microscopic analyses show that the portion sequence NTT-MiPM is a CPP/PTD. Nevertheless, these data do not exclude the possibility that the core protein could be implicated in the translocation process. At first sight, the N-terminal region appears to be a tail-like unstructured amino acid chain containing a basic and an acidic region separated by hydrophobic and polar residues. Of note, these features can also be found, for instance, in RxLR oomycete effectors (Whisson et al., 2007). Assuming that the histidine (H) residues of this region are protonated once in the apoplast, the sequence “RRHHR” shares similarities with some cationic CPP/PTDs. In the HIV transactivator protein (Tat), the unfolded transduction domain “GRKKRRQRRRQ” was one of the first CPP/PTDs described (Vivès et al., 1997). The NTT of the capsid protein from the phytovirus Brome Mosaic Virus (BMV) “RAQRRAAARR” is likewise an arginine-rich (R) motif capable of cell entry in barley protoplasts (Qi et al., 2011). NTT-MiPM is shorter, but the surrounding amino acids should be integrated into the model. Indeed, rational design and molecular evolution experiments on CPP/PTD have highlighted some strategic positions of polar and hydrophobic residues in a peptide or small protein. In this configuration, CPP/PTD peptides that contain 2–6 arginine residues can be taken up more efficiently than HIV-Tat (Nishimura et al., 2008; Smith et al., 2008; Marks et al., 2011; Milletti, 2012; Gautam et al., 2013). In plant antifungal defensins, the motif “RGFRRR” located in a surface exposed loop of the core protein is required for fungal cell entry (Sagaram et al., 2013). The hydrophobic and polar residues “VQA” of NTT-MiPM located between the basic and acidic motif might determine the mechanism of translocation across the host plasma membrane.

Our microscopic analyses indicate that ^*^MiPM^−SP^ can trigger the endocytosis pathway, which raises the intriguing question of how MiPM translocates across the plant plasma membrane. One hypothesis is that MiPM enters the host cells using a receptor-mediated endocytosis. Pathogens can exploit the function of host receptors on the plasma membrane and thereby the endocytosis process to allow cell entry (Ewers and Helenius, 2011; Grove and Marsh, 2011; Cossart and Helenius, 2014). For example, HIV-Tat induces a specific endocytosis pathway called macropinocytosis at the plasma membrane, mediated by an interaction with the specific chemokine receptor CXCR4 (Tanaka et al., 2012). In barley protoplasts, the use of macropinocytosis inhibitors has demonstrated the existence of a similar uptake mechanism acting on the CPP/PTD from the phytovirus BMV capsid protein (Qi et al., 2011). Some specific immune receptors can be targeted. The bacterial effector flagellin interacts with the membrane receptor FLS2 after its endocytosis (Robatzek et al., 2006; Nathalie Leborgne-Castel and Bouhidel, 2014; Hohmann et al., 2017). Other plant cellular pathways could be investigated. Interestingly RD21B localizes in different subcellular compartments such as the apoplast, endoplasmic reticulum (ER), trans-Golgi network (TGN)-derived endosomes and vacuoles (Hayashi et al., 2001; Yamada et al., 2001; Bozkurt et al., 2011; Gu et al., 2012; Lampl et al., 2013; Rustgi et al., 2017). Although it is not clear whether RD21B can re-enter the cell by endocytosis, RD21B appears to use the endocytosis pathway to traffic in and out of the cell. It is tempting to speculate that MiPM could interact a protein like RD21B to borrow an endocytosis pathway. Moreover, the specific lipid and proteoglycan composition of the plasma membrane, termed its ZIP code (also known as phospholipid code) contributes to cell identity and signaling (Walley et al., 2013; Baxter et al., 2015). For instance, the specific binding of phosphatidylinositol-3-phosphate [PI(3)P] is necessary for the cell entry of certain RxLR oomycete effectors (Whisson et al., 2007; Kale et al., 2010; Sun et al., 2013). We observed the accumulation of NTT-MiPM on the plasma membrane. Some CPP/PTDs bind and cluster specifically with phospholipids or proteoglycans of the cell membrane to induce endocytosis (Belting, 2003; Fischer et al., 2005). Thus the binding of phospholipids or other membrane components cannot be excluded.

The membrane status responds to developmental and environmental variations. For instance, the distribution of some phospholipids is heterologous and clearly delineated within the Arabidopsis shoot apical meristem (Stanislas et al., 2018). In another example, the plant membrane lipid composition varies in contact with pathogens and contributes the activation of plant immunity (Zoeller et al., 2012; Walley et al., 2013). The cell identity and the physiological context matters and might tune cellular entry of molecules through changes in phospholipid composition and content. As previously mentioned, some CPP/PTDs can interact specifically with a variety of lipids. In addition, the infectious context activates oxidoreductases on the plasma membrane at early time points in infection. A local production of reactive oxygen species leads to specific lipid oxidation (Zoeller et al., 2012). Hence, the cell entry of certain CPP/PTDs is enhanced with an elevated level of oxidized lipids (Wang and Pellois, 2016; Wang et al., 2016). At another potential regulatory layer, the pH of the root apoplast varies with circadian rythms and greatly depends on the tissue and on environmental conditions such as abiotic and biotic stresses (Geilfus, 2017). Biochemical models suggest that slight environmental alkalinization induces lipid deprotonation and enhances HIV-Tat interaction (Herce et al., 2014). In this study, we incubated the recombinant fluorescently labeled ^*^MiPM^−SP^ with Arabidopsis root tips free of pathogen infection. Considering the parameters previously mentioned, the efficiency of the cell entry of secreted CPP/PTDs, such as MiPM, might vary with the physiological state of the plant.

### MiPM escapes the endosomal trap

After cell entry, another concern for pathogenic secreted proteins is the endosomal trap. In animals, early endosomes can turn within minutes into late endosomes and then lysosomes (Huotari and Helenius, 2011). In this later situation, the cargo can undergo degradation by the proteasome. In plants, vesicles from the *trans*-Golgi network act as an early endosome, and the endocytic cargo is delivered to the vacuole or the proteasome (Paez Valencia et al., 2016). It is remarkable to detect MiPM^−SP^ in vesicles-like structures as well as in the nucleus, which highlights some ability of the protein to escape the endocytosis pathway. It is not clear whether NTT-MiPM shares this particular characteristic, since the peptide seems to accumulate in vesicle-like structures or in the vacuole. Mechanistically, endosomal escape could be initiated by the protein itself or by the recruitment of host protein partners (Grove and Marsh, 2011; Bissig and Gruenberg, 2013). In the first option, the molecular mechanism has been described in viral proteins that contain a pH-dependent membrane active peptide (Erazo-Oliveras et al., 2012; Lönn et al., 2016; Akishiba et al., 2017). Other potential mechanisms might involve a change in the membrane lipid composition during endosome maturation to activate the endosolysosomal properties of the protein (Appelbaum et al., 2012). Alternatively, host proteins can mediate the protein sorting from endosomes (Byk et al., 2016; Heucken and Ivanov, 2017). The ubiquitin-proteasome system is an active regulator of endosome protein sorting (Clague et al., 2012) and some human viruses use it to reach the host cytoplasm (Liu et al., 2013; Su et al., 2013; Banerjee et al., 2014; Byk et al., 2016; Rudnicka and Yamauchi, 2016). We previously mentioned that GmCSN5 interacts with MiPM in the nucleus, as shown by BiFC. Whereas CSN5 occurs predominantly in the CSN complex in the nucleus, other CSN5 subcomplexes exist in the cytoplasm, whose roles and protein partners remain poorly defined (Gusmaroli et al., 2004; Wei et al., 2008; Schwechheimer and Isono, 2010; Pick and Bramasole, 2014). A previous study has demonstrated that HsCSN5 is involved in vesicle trafficking (Liu et al., 2009). The AP-MS interactome of HsCSN5 has identified a conserved eukaryotic vesicle trafficking protein closely related to the sorting nexin, dynein, myosin, or clathrin family (Bennett et al., 2010). In addition, the conserved JAMM/MPN domain of AMSH, an endosome-associated ubiquitin peptidase, is determinant for the vesicle sorting of many proteins in plants (Isono et al., 2010) and in animals (McCullough et al., 2004). In the biological context of our BiFC, MiPM did not traffic through the endocytic pathway, and both proteins are produced within the cell, increasing their chance of interaction in the nucleus. The possibility that CSN5 regulates the endosomal sorting of MiPM can be considered.

## Conclusion

In our biochemical approach, some previously characterized CSN5 interaction proteins are described such as the CSN complex, CC1/RBM39 and APC2. We also identified the novel nematode-secreted protein MiPM. In this study, we focused on the MiPM^−SP^-GmCSN5 interaction. Our data from affinity-based systems have identified differences, but interestingly, a number of isolated proteins in the two interactomes overlap (near 11%). Mechanistically, our knowledge about these interactions and their biological significance is fragmentary. The generation of transgenic Arabidopsis plants expressing MiPM would be essential for understanding its role in parasitism. The characterization of MiPM secretion during parasitism is determinant as well. Moreover, it is of considerable interest to fill the gaps in our understanding of the mechanism of translocation involving MiPM protein. Remarkably, preliminary biophysical analyses showed that MiPM is a stable protein at various pH values (data not shown) and can support some biochemical modifications, such as the conjugation of a fluorescent probe. Some proteomic approaches conjugated with proximity labeling, such as click chemistry, have proven to be useful and efficient for identifying proteins implicated in a specific endocytosis pathway (Tanaka et al., 2012; McFedries et al., 2013). Thus, the future identification of MiPM interaction proteins during cell entry by such approaches might contribute to a better understanding of its mode of action.

## Availability of materials

The authors confirm that all data underlying the findings are included in this published article and are fully available without restriction.

## Author contributions

Conceptualization: CB; Data acquisition: CB, F-XG, AM (MS technical support), EB (computational analysis of APEX-GmCSN5); Methodology: CB and F-XG; Resources: CB, EA, and MG; Writing—original draft and editing: CB and F-XG. CB, F-XG, AM, EA, and MG reviewed the paper. All authors read and approved the submitted version.

### Conflict of interest statement

The authors declare that the research was conducted in the absence of any commercial or financial relationships that could be construed as a potential conflict of interest.

## Abbreviations

APEX: engineered ascorbate peroxidase
AP-MS: affinity-purification—mass spectrometry
CSN: COP9 signalosome
GST: Glutathione S Transferase
HR: hypersensitive response
NTT: N-terminal tail
PLCP: papain-like cysteine protease
PTD: protein transduction domain
RKN: root-knot nematode
SP: secretory peptide.
