# Supplementary material for: Meloidogyne incognita PASSE-MURAILLE (MiPM) Gene Encodes a Cell-Penetrating Protein That Interacts With the CSN5 Subunit of the COP9 Signalosome
Source: Front Plant Sci. 2018 Jun 26;9:904. doi: 10.3389/fpls.2018.00904 (PMC6029430; doi:10.3389/fpls.2018.00904)
Supplement: Supplementary file 6 [file Presentation_1.pdf]

Supplementary information for

Meloidogyne incognita PASSE-MURAILLE (MiPM) gene encodes a cell-penetrating protein that interacts with the CSN5 subunit of the COP9 signalosome

Authors

Caroline Bournaud[1¶*], François-Xavier Gillet[1¶*], André Melro Murad[1], Emmanuel Bresso[2], Erika Valéria Saliba Albuquerque[1&], Maria Fatima Grossi-de-Sá[1,3&*]

*** To whom correspondence should be addressed.**

Caroline Bournaud: bournaudcaroline8@gmail.com
François-Xavier Gillet: francois-xavier.gillet@hotmail.fr
Maria Fatima Grossi-de-Sá: fatima.grossi@embrapa.br

This pdf includes:

- Supplemental result and discussion
- Supplemental Figures and legends S1-S9
- Supplemental material and methods
- Supplemental references

Other supplemental material includes:

- Table S1-S4
- Video S1

SUPPLEMENTAL RESULT AND DISCUSSION

Design investigation and model APEX-GmCSN5

The APEX system is based on an engineered enzyme, soybean ascorbate peroxidase, optimized to enhance its catalytic activity >25-fold over the native ascorbate peroxidase (Lam et al., 2015; Martell et al., 2012). The APEX-protein of interest fusion protein catalyzes a biotin-phenol (BP) substrate into a phenoxy radical that selectively and covalently tag nearby proteins (with nanometer spatial resolution and fast reaction times) in living cells with biotin (Lobingier et al., 2017). The **Figure S2A** summarizes the mechanism of proximity-based labeling using APEX, taking as an example APEX-GmCSN5 integrated in the CSN complex. Noteworthy, we noticed several concerns that might interfere with the functionality of APEX-GmCSN5 both in proximity-based labeling and affinity purification approaches:

(**1**) APEX might alter by steric hindrance the GmCSN5 folding, activities as well as its protein interaction. The design of a suitable linker was thus a prerequisite to avoid these limitations. We focused on a flexible rather than a rigid linker since it is more convenient when the joined moieties require a certain degree of movement or interaction (Chen et al., 2013).

(**2**) The length of the flexible linker might be considered to avoid artifactual protein labeling. It has been estimated that the distance compatible for efficient APEX labeling would be less than 20 nm (Rhee et al., 2013; Hung et al., 2014). Interestingly the human CSN5 and its ortholog in soybean share high amino acid identity (67%) (**Supplementary Figure S1**) and the structure of HsCSN5 had been solved within the conserved CSN complex (PDB ID: 4D10; Lingaraju et al., 2014). The structure of the soybean ascorbate peroxidase is also available (PDB ID: 1OAF; Sharp et al., 2003). Hence, we took advantage of these elements to model by homology the APEX-GmCSN5 within the human CSN complex (**Supplementary Figure S2A-B; video S1**). We designed a flexible glycine-rich linker of 20 amino acid residues (~8,4 nm) that separates APEX from the mass centre of GmCSN5 and other CSN subunits in an average length of ~10±1,6 nm.

(**3**) The flexible linker might increase the ability of APEX and GmCSN5 to interact together. We computed a molecular dynamics simulation to evaluate the interference of the two linked proteins over 100 ns (**Supplementary Figure S2C**). None potential energy was detected between APEX and GmCSN5 and both proteins have individually a lower potential energy with water. This indicates that the interaction between GmCSN5 and APEX is negligible.

(**4**) Finally, we attempted to determine the position of the two strep-tag II linked to GmCSN5 in our 3D model (**Supplementary Figure S2A-B**; **video S1**). The N-terminal strep-tag II is solvent exposed and located on the catalytic side of the CSN, which is determinant for the tethering of the tag to streptactin substrate during purification. Owing to the lack of structural data in the C-terminal part of HsCSN5, the second strep-tag II was not positioned. According to the model previously proposed (Lingaraju et al., 2014), we postulate that the strep-tag II is accessible for the binding of the streptactin. Altogether, our computational approach suggests that the design of APEX-GmCSN5 is suitable to carry out affinity-based experiments.

Investigating proximity labeling *in planta* using APEX-GmCSN5

We have examined the feasibility of the APEX system for identifying GmCSN5 protein partners in plants. Despite numerous repetitions and modifications in our experimental design, we did not identify any peptides from these experiments. We present here the main results obtained and concerns encountered.

Our experimental setup was similar to those developed in animal cells, with slight adaptations for the delivery and the concentration of chemical inducers in plants (**Supplementary Figure S3B, see material and methods**) – (Hung et al., 2016; Hwang and Espenshade, 2016; Reinke et al., 2017). We initially focused on tobacco leaves instead of roots for practical reasons and simplicity. We first assessed biotinylation of endogenous proteins in wild type (WT) plant versus transgenic lines expressing constitutively APEX-GmCSN5. Leaves were treated with BP and hydrogen peroxide (H_2O_2) before the extraction and purification of biotinylated proteins using streptavidin-coated magnetic beads (**Supplementary Figure S3B**). Working with identical protein quantities, the amount of purified proteins was slightly higher from transgenic line (11.11) than WT (**Supplementary Figure S3C**). This result suggests that APEX contributes to the biotinylation of endogenous proteins. Under conditions mentioned above, we next examined the biotinylation pattern in treated leaves of tobacco transgenic line with or without BP treatment (**Supplementary Figure S3D**). Using a streptavidin-conjugated horseradish peroxidase (HRP), the blot revealed a consistent enrichment of the biotinylated proteins after treatment with BP and H_2O_2. Two intense bands were detected in both samples from total soluble protein, which remind the endogenous biotinylated proteins MCCA (3-methylcrotonyl CoA carboxylase; 78 kDA) and BCCD (Biotin carboxyl carrier protein domain; 30 kDA) (Qi and Katagiri, 2009). However, these proteins were poorly accumulated in the elution fractions. Having confirmed a distinct biotinylation pattern, we next examined the detection of APEX-GmCSN5 on these eluted samples by western blot using a strep-tag II antibody (**Supplementary Figure S3D**). Surprisingly, we detected APEX-GmCSN5 from the sample devoid of BP treatment. This observation enters in resonance with a study showing that the strep-tag II can bind streptavidin (Korndörfer and Skerra, 2002; Schmidt and Skerra, 2007). Hence we postulated that the purification of biotinylated proteins gave an enrichment of APEX-GmCSN5 in the samples. Notably this later signal was weak in the negative control. Numerous additional bands were also detected in a window range *c.* 50 – 80 kDa bordering APEX-GmCSN5. We also observed that the intensity of the signal enhanced with increased BP concentration (up to 5 mM – data not shown). This result suggests that APEX-GmCSN5 might form covalent bonds with other proteins. Its detection at lower molecular weight suggests that APEX-GmCSN5 might be cleaved. This atypical pattern observed by immunodetection of APEX-GmCSN5 retained our attention. The protein was detected at higher and lower molecular weight than APEX-GmCSN5. The electrophoresis pattern of the proteins is more closely related to the detection of bands than a smear. We first thought that the antibody might expire causing the detection of non-specific proteins. However we successfully used it to detect APEX-GmCSN5 from leaves of transgenic *N. tabacum* lines compared to the WT (**Supplementary Figure S3E**). To test the hypothesis that higher molecular weight could result to intermolecular disulfide bonds, the protein samples were treated with reducing agent (Dithiothreitol, DTT – up to 100 mM). We did not observe any differences with or without DTT treatment. As previously reported (Hung et al., 2014), we postulate that APEX-GmCSN5 might be covalently cross-linked with other proteins in its entire or cleaved form and undergone some break damages due to the external chemical reagents (i.e BP, quenchers). Regarding the complex secondary metabolism in plants, some metabolites could also be a source of post-translational modifications. Moreover, the low abundance of purified biotinylated proteins increases our difficulties to identify peptide by mass spectrometry in these experiments.

Despite the rising popularity of APEX technology, several studies pointed out certain weaknesses such as H_2O_2 toxicity and the mode of delivery of chemical inducers, which may require to genetically or manually manipulate *in vivo* systems (Chen et al., 2015; Branon et al., 2017; Reinke et al., 2017). Whether APEX system appears clearly attractive in

mammalian cells or other animal systems, the proximity-dependent biotin identification (BioID) method has also proved effective results (Roux et al., 2012; Branon et al., 2017). BioID has recently brought important cues for isolating and identifying relevant protein-protein interactions in rice protoplasts (Lin et al., 2017).

SUPPLEMENTAL FIGURES

FIGURE S1

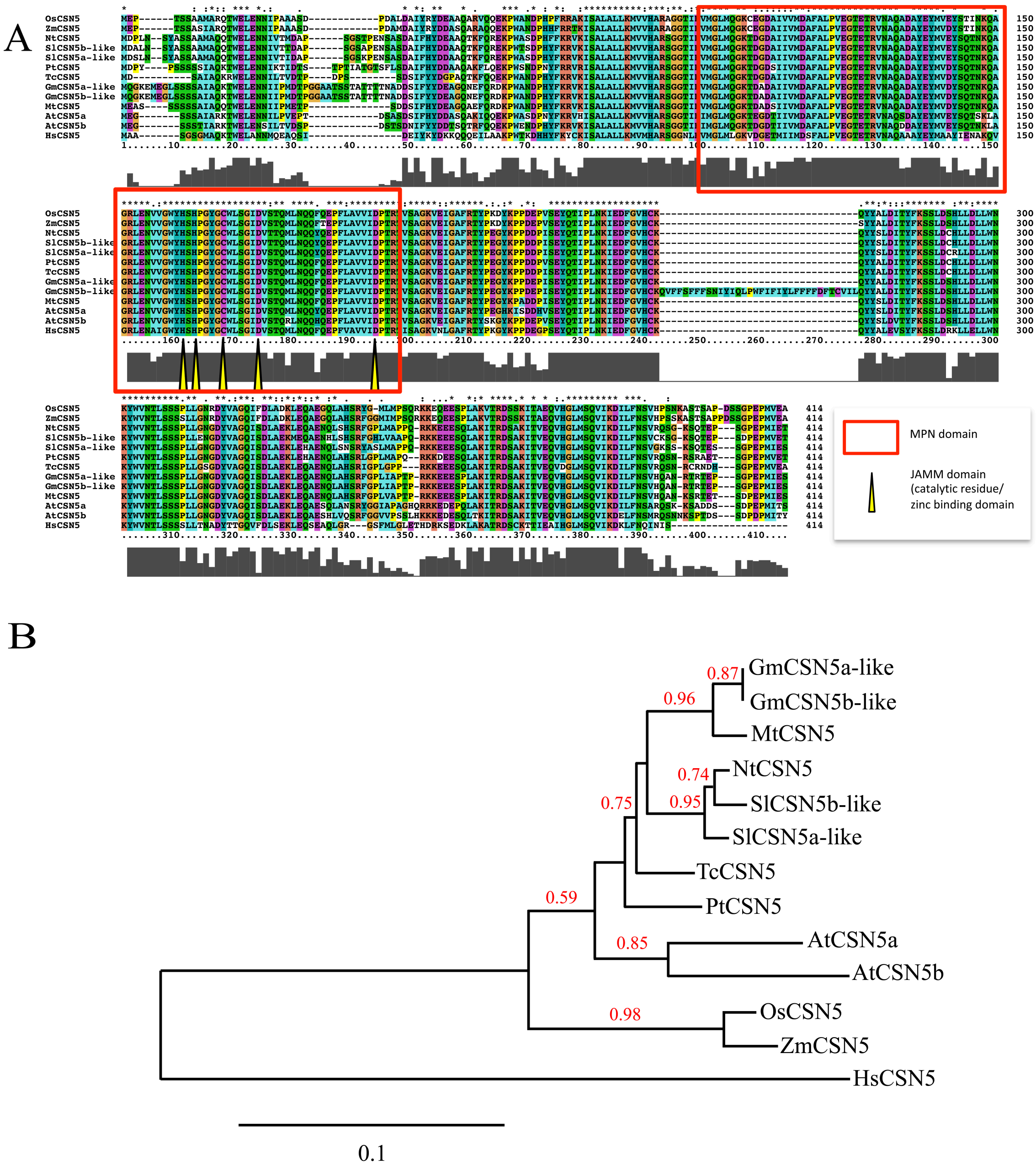

Figure S1. GmCSN5 is highly conserved among its plant homologs. (A) Comparison of the CSN5 amino acid sequences of different plant species and human performed using Clustal X v2.1. Arabidopsis displays two isoforms of CSN5 and were used as reference proteins. Both species, *Glycine max* and *Solanum lycopersicum*, were predicted to possess two CSN5 copies compared with other representative plant species (Jin et al., 2014). AtCSN5a: isoform CSN5a from *Arabidopsis thaliana* (Q8LAZ7); AtCSN5b: isoform CSN5b from *Arabidopsis thaliana* (Q9FVU9); GmCSN5a-like: ortholog of the isoform AtCSN5a from *Glycine max* (Glyma.04G075000.1); GmCSN5b-like: ortholog of the isoform AtCSN5b from *Glycine max* (Glyma.06G076000.1); HsCSN5: CSN5 from *Homo sapiens* (Q92905); MtCSN5: CSN5 from *Medicago truncatula* (G7JCC9); NtCSN5: CSN5 from *Nicotiana tabacum* (XP_016434486);

OsCSN5: CSN5 from *Oryza sativa subsp. japonica* (Q8H936); PtCSN5: CSN5 from *Populus trichocarpa* (B9ILG7); SlCSN5a: isoform CSN5a-like from *Solanum lycopersicum* (K4C935); SlCSN5b: isoform CSN5b-like from *Solanum lycopersicum* (Q9FR56); TcCSN5: CSN5 from *Theobroma cacao* (K09613); ZmCSN5: CSN5 from *Zea mays* (B4FUK9). The conserved MPN domain (red box) and JAMM residues (yellow arrows) are indicated. **(B)** Phylogenetic tree of complete CSN5 protein sequences from representative plant species shown in **(A)** illustrates the high degree of conservation of this protein in the plant kingdom. The phylogenetic tree was built by Muscle alignment and generated by the maximum likelihood (ML) method. The branches were supported with 100 bootstrap replicates (shown > 50%). The distance from the branches represents mutations per amino acid, as shown by the scale (http://www.phylogeny.fr/; Dereeper et al., 2008).

FIGURE S2

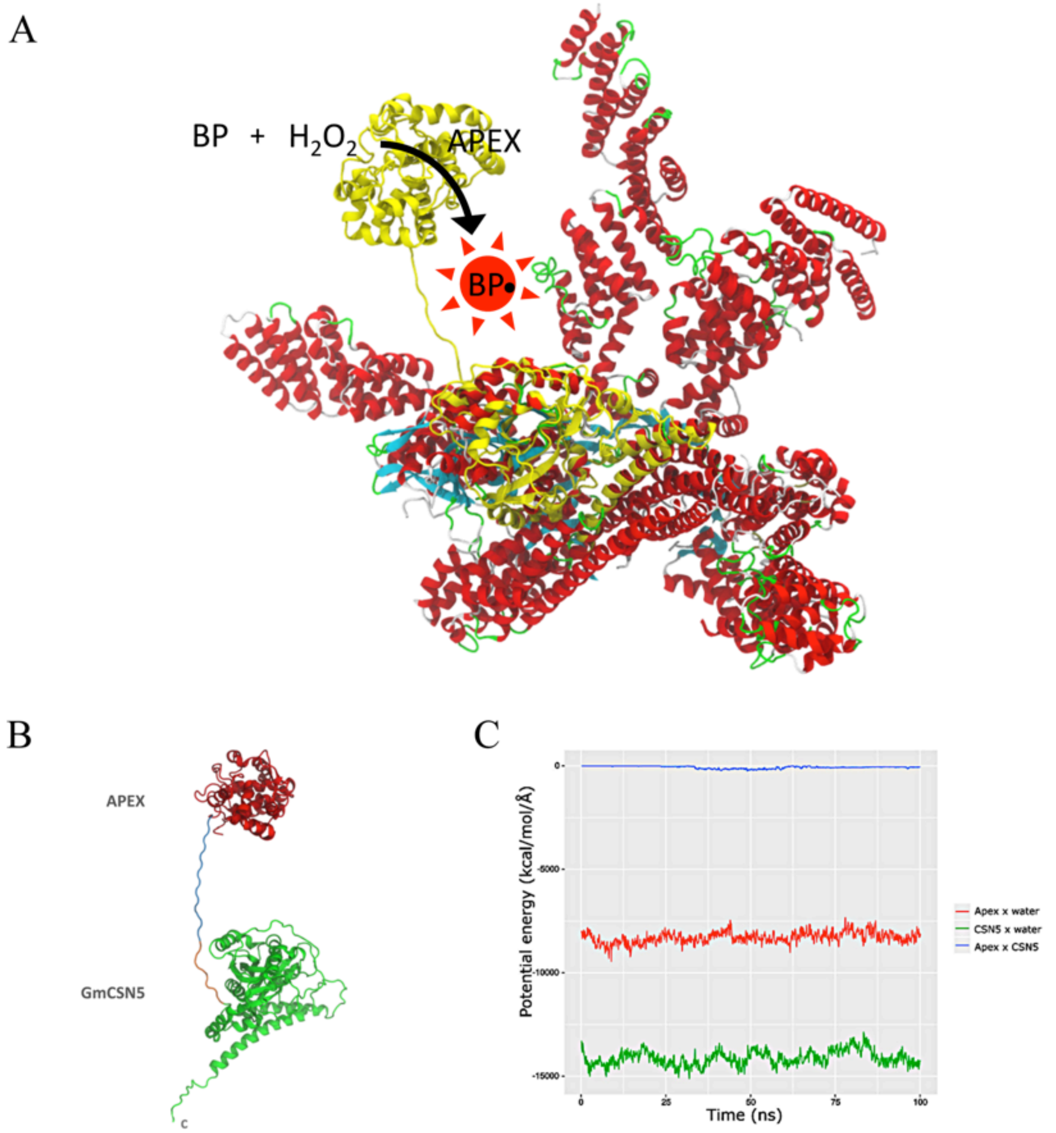

Figure S2. APEX-GmCSN5 3D model and molecular dynamic simulations between APEX and GmCSN5. (A) Illustration of proximity-based labeling applied to APEX-GmCSN5 integrated in the CSN complex. APEX catalyzes the reaction between the biotin-phenol (BP) hydrogen peroxide (H_2O_2) leading to the generation of biotin-phenoxy radicals (BP•), which covalently label GmCSN5-interacting proteins in a proximity-dependent manner. APEX-GmCSN5 (yellow) was built by homology modeling and integrated in the human CSN complex (red). The overlap of the secondary structures between HsCSN5 (red) and GmCSN5 (yellow) is shown. **(B)** Cartoon representation of APEX-GmCSN5. APEX and GmCSN5 were built by homology modeling (PDB ID: 1OAF and 4D10 respectively). APEX: red; flexible linker: blue; N-terminal strep-tag II: gold; GmCSN5: green. **(C)** Pair interaction energies measurement obtained by molecular dynamics simulation. The degree of interaction between the moieties of APEX and GmCSN5 is assessed by the measurement of the potential energy. For the comparison, the potential energy between APEX and water and GmCSN5 and water is shown.

FIGURE S3

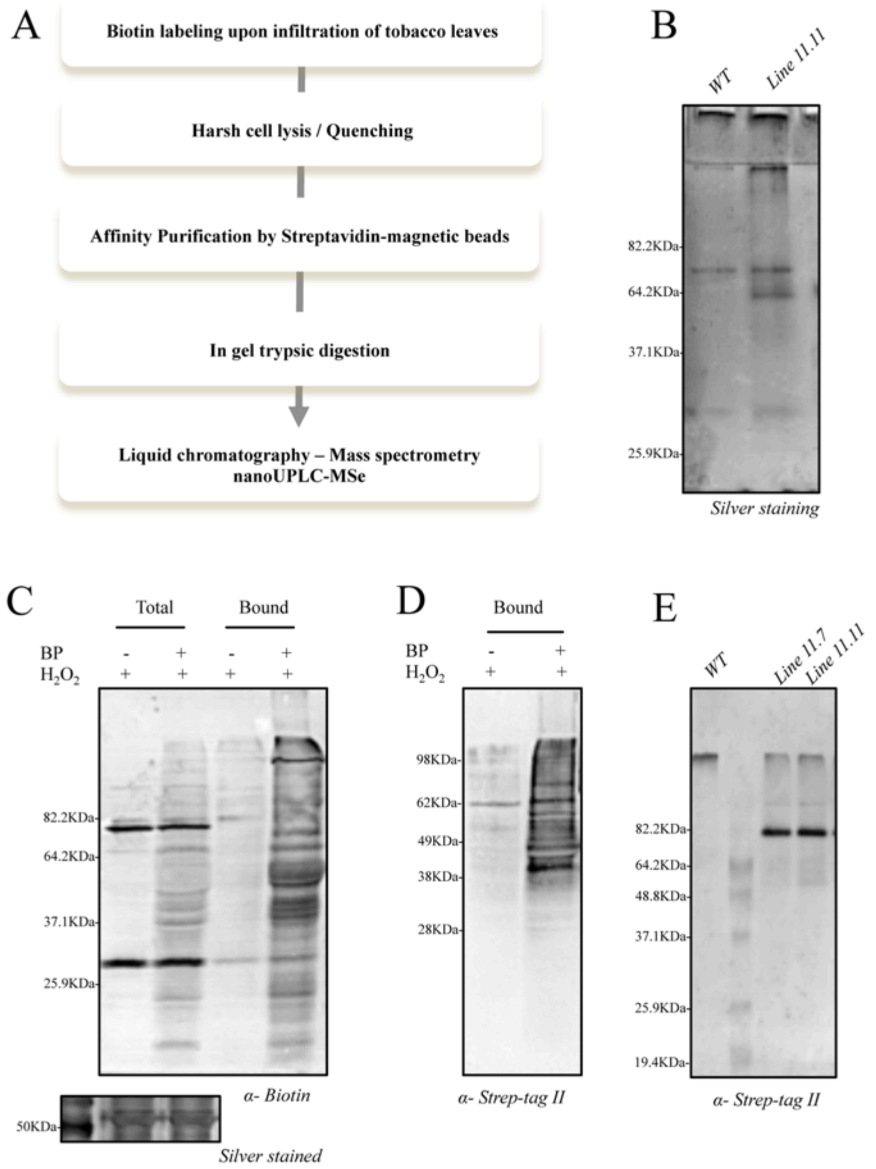

Figure S3. Our preliminary test for APEX proximity labeling is not effective to capture GmCSN5 partners in transgenic *N. tabacum* leaves. **(A)** Workflow of APEX system for characterizing GmCSN5-interacting proteins in plant system. **(B)** Silver stain analysis indicates that an enhanced accumulation of streptavidin-purified proteins in transgenic line (11.11) overexpressing APEX-GmCSN5 in response to chemical treatments compared with the WT plant. Purified proteins were obtained from leaf samples infiltrated with 5 mM BP for 30 min and then with 1 mM H_2O_2 for 1 min. **(C)** In equal amount of extracted soluble total proteins from the transgenic sample (11.11), an increased biotinylation of endogenous proteins is only observed in response to BP/H_2O_2. Protein fractions collected prior to purification (**Total**) and those that bound streptadivin-coated magnetic beads (**Bound**) were visualized by western blotting using streptavidin-conjugated HRP. **(D)** Whether APEX can covalently label endogenous proteins in tobacco transgenic line (11.11), the integrity of APEX-GmCSN5 is compromised by the presence of chemical inducers. Eluates (**Bound**) were analysed by western blotting using the Strep-tag II antibody. **(E)** Total soluble proteins from leaves of WT and transgenic lines (11.7 and 11.11) were extracted and analysed by western blot using the Strep-tag II antibody.

FIGURE S4

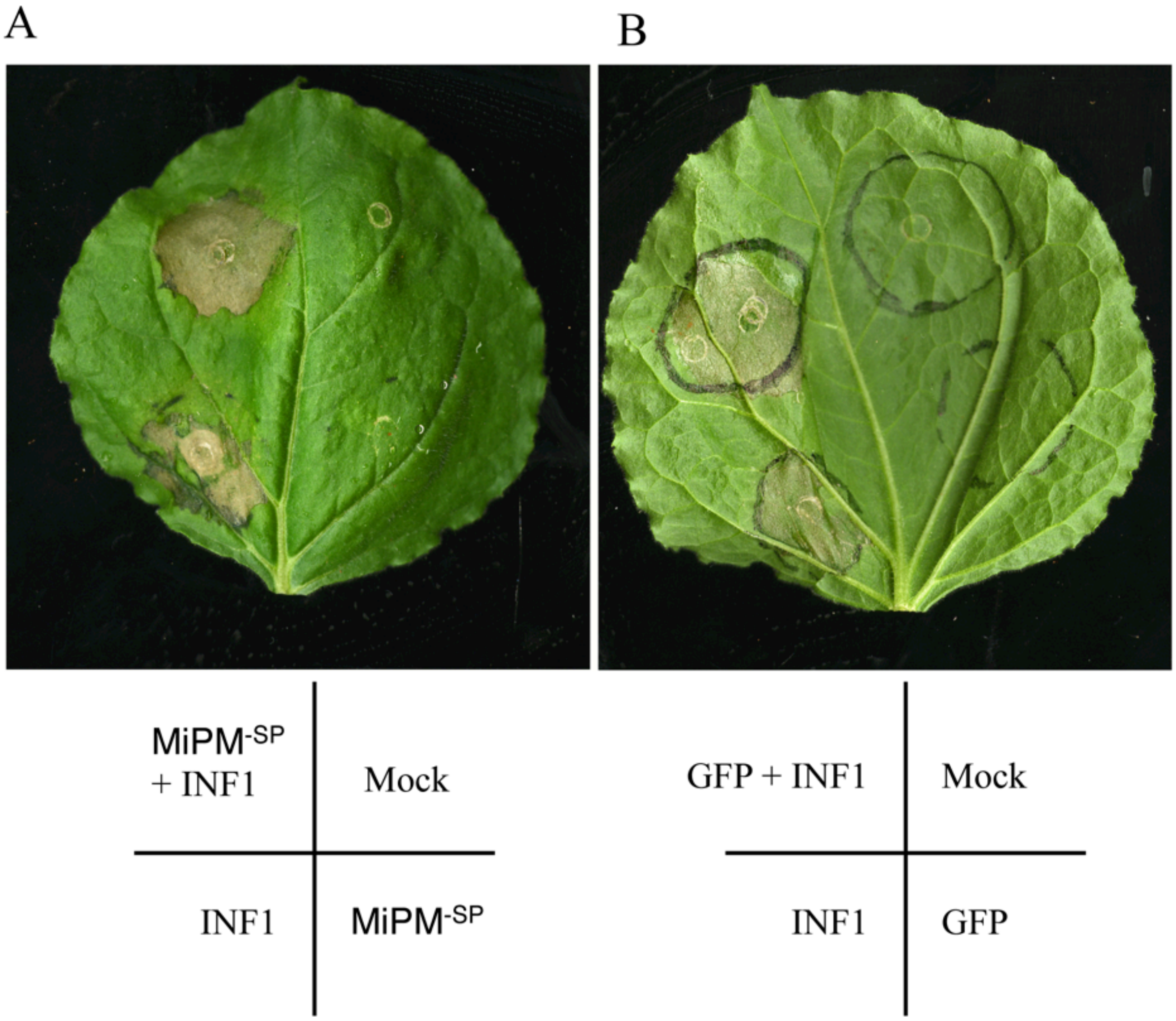

Figure S4. Ectopic expression of MiPM^{-SP}protein does not suppress the cell death induced by the elicitor INF1. (A) Leaves of *N. benthamiana* were agroinfiltrated with buffer (Mock) or with agrobacterium for heterologous expression of MiPM^{-SP} gene solely or co-infiltrated after 24 h with agrobacterium expressing the oomycete INF1 gene. **(B)** GFP gene was used as negative control. Representative images of cell death phenotypes were acquired after 6 days after inoculation.

FIGURE S5

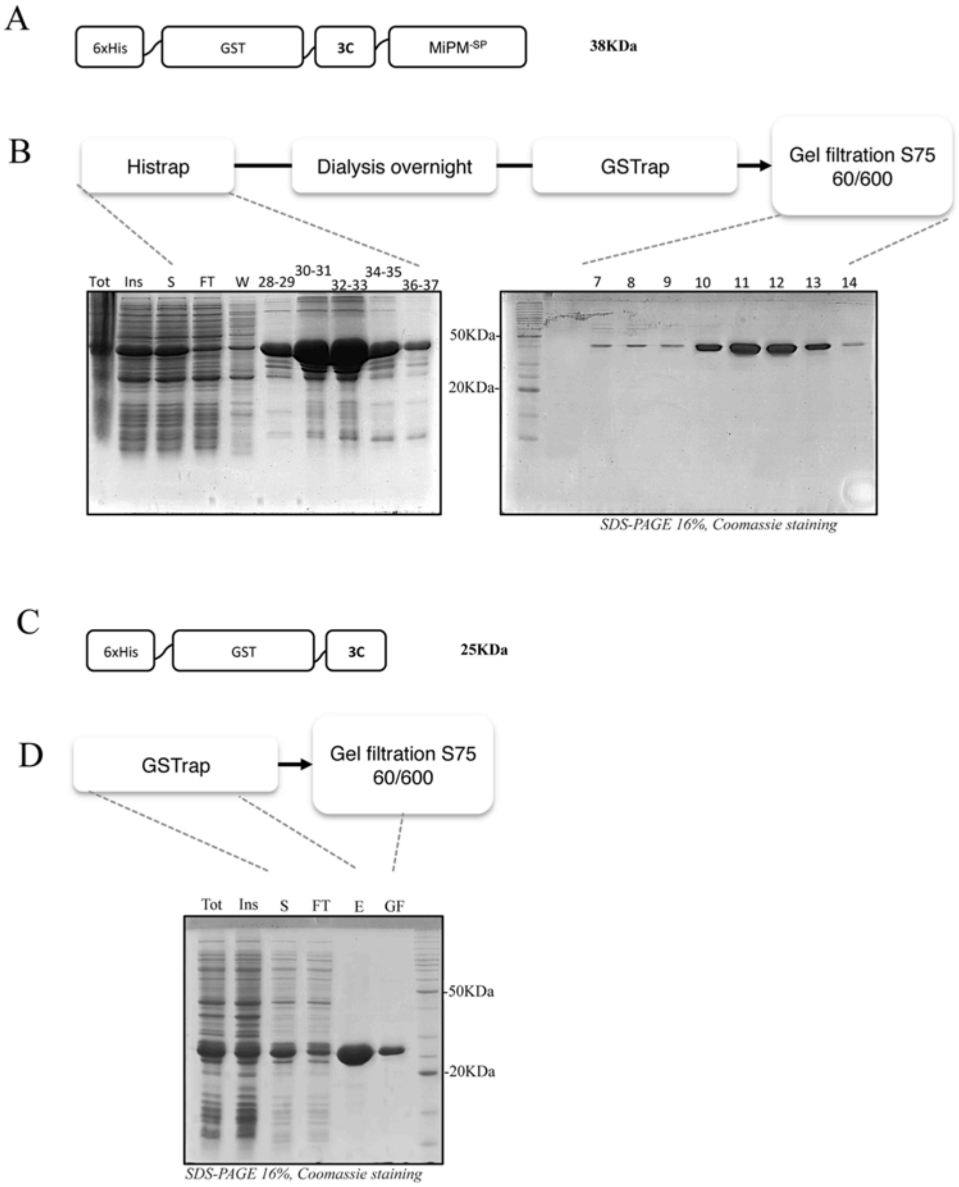

Figure S5. Purification of GST-MiPM^{-SP}. (A) Schematic representation of the chimeric protein GST-MiPM^{-SP}. **(B)** The left panel describes the purification of the native GST-MiPM^{-SP} by immobilized metal affinity chromatography (IMAC) by using the 6xHis tag and HisTrap columns. The protein was isolated by gel filtration (right panel). **(C)** Schematic representation of the GST protein. **(D)** The purification of the native GST protein by GST-trap system. The protein was isolated by gel filtration. Tot: Total; Ins: Insoluble; S: Soluble; FT: Flow-through; W: Wash; numbered lanes: elution fractions; E: elution

FIGURE S6

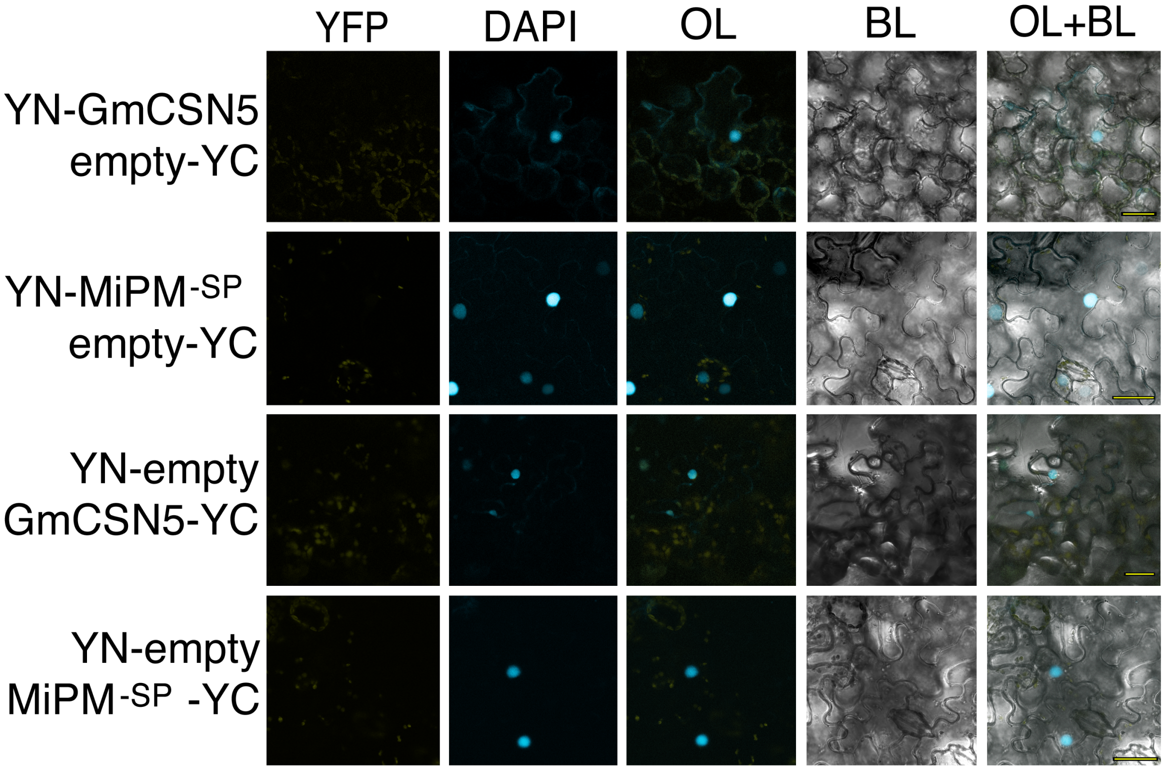

Figure S6. No YFP fluorescence was observed following the co-expression of empty YN and YC constructs with MiMP^{-SP} or GmCSN5 constructs in *N. benthamiana* leaves. After co-bombardment of the combination YN-GmCSN5/YC, YN/GmCSN5-YC, YN-MiPM^{-SP}/YC and YN/MiPM^{-SP}-YC, no noticeable YFP fluorescence was detected in the nucleus of *N. benthamiana* leaf epidermal cells, as confirmed by the DAPI stain. All images were taken 24-48h after bombardment across two independent biological experiments BL: bright light; OL: overlay.

FIGURE S7

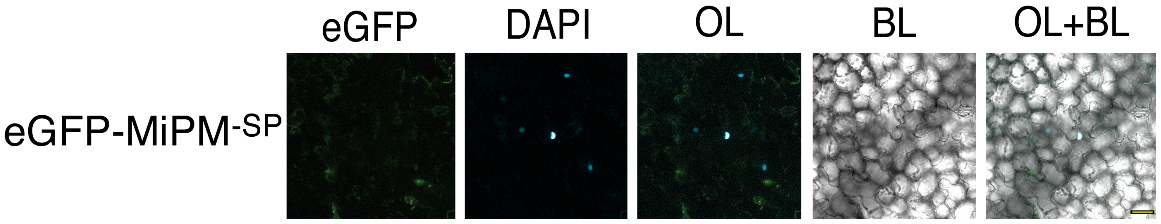

Figure S7. No GFP signal was detected following the expression of eGFP-MiPM^{-SP} in the bombarded *N. benthamiana* leaves. After the bombardment with the construct eGFP-MiPM^{-SP}, no GFP fluorescence was observed in the nucleus of *N. benthamiana* leaf epidermal cells, as confirmed by the DAPI stain. All images were taken 24-48h after bombardment across two independent biological experiments. BL: bright light; OL: overlay.

FIGURE S8

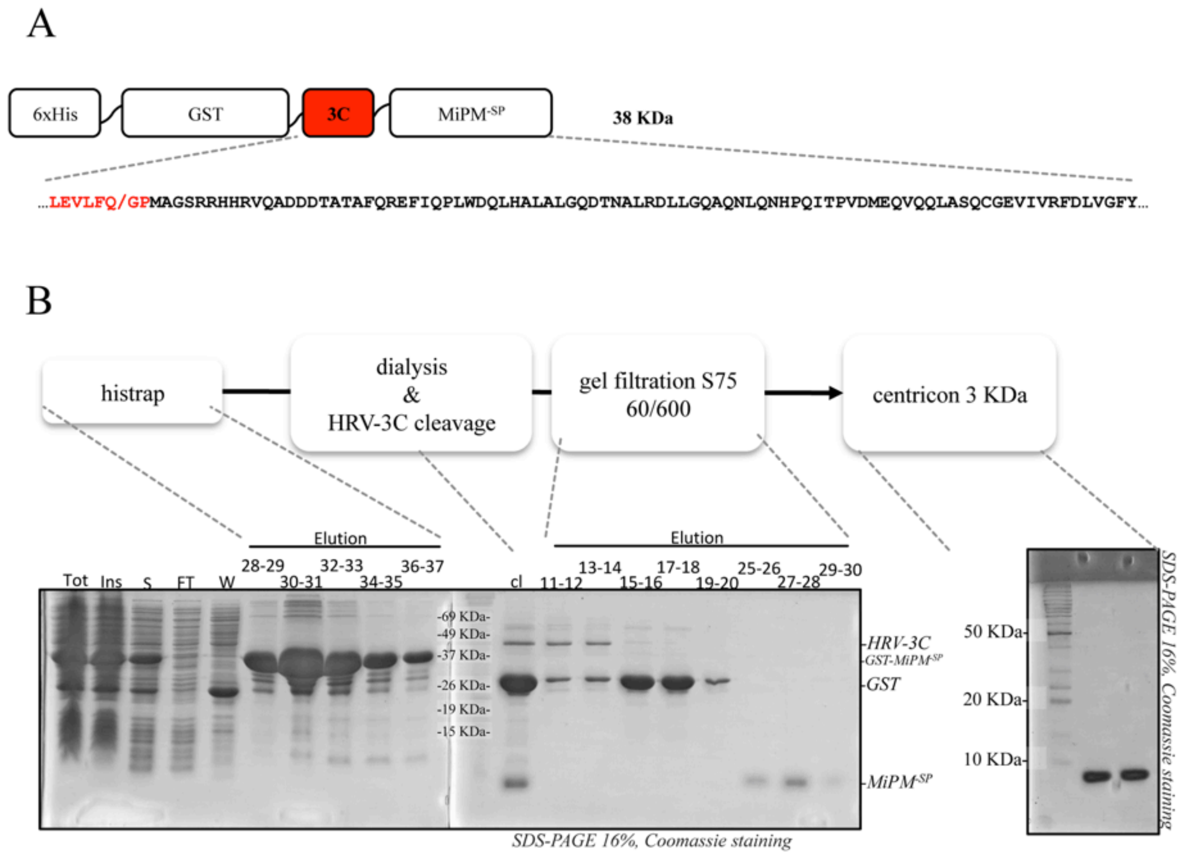

Figure S8. Purification of MiPM^{-SP}. (A) Schematic representation of the chimeric protein MiPM^{-SP}. The full sequence of MiPM^{-SP} is represented. The "/" indicates the HRV-3C (or Prescission) proteolytic cleavage site, and the sequence recognized by HRV-3C is shown in red. **(B)** The left panel describes the purification of native GST-MiPM^{-SP} by immobilized metal affinity chromatography (IMAC) by using the 6xHis tag and His-trap columns. The protein was cleaved overnight using Prescission protease and isolated by gel filtration (right panel). Tot: Total; Ins: Insoluble; S: Soluble; FT: Flow-through; W: Wash; numbered lanes: elution fractions.

FIGURE S9

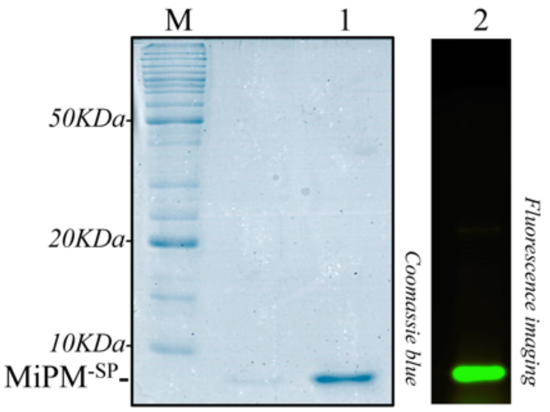

Figure S9. Intactness of MiPM^{-SP} protein tagged with Alexa488 probe before use for cell entry assay in Arabidopsis root tips. The purified (lane 1) and concentrated (lane 2) recombinant protein were separated by 16% SDS-PAGE and visualized by Coomassie blue staining (left panel). The fluorescent detection (right panel) of the MiPM^{-SP} protein fused to the probe Alexa488 was performed by scanning the SDS gel with a Typhoon scanner imaging system (excitation 488 nm; emission 520 nm).

Video S1. 3D structure of APEX-GmCSN5 modeled by homology in the human CSN complex

MATERIALS AND METHODS

Model - Lacking experimental solved 3D structure of the GmCSN5 protein, the 3D model was built using homology modeling. The first step consisted to retrieve the most similar protein from the protein data bank (PDB, https://www.rcsb.org/), a database of known structures. The CSN5 template was achieved from the human CSN5 (PDB ID: 4D10; Lingaraju et al., 2014) and the APEX structure was extracted from PDB ID: 1OAF (Sharp et al., 2003). Once the CSN5 template and the APEX structure were identified, the homology model was built using MODELLER with its default settings (Webb and Sali, 2017). Loops were optimized by the MODELLER automatic loop refinement method. Three different models were built and the one with the best DOPE score was retained for optimisation with a 100 ns MD simulation.

Molecular dynamics simulation - From the refined model previously obtained, we instigated its behaviour in a physiological medium. The protein was embedded in a 110x175x140 Å box with 245,595 TIP3P explicit water molecules. Five sodium ions were added to ensure the electrostatic neutrality. The NAMD program version 2.9 (Phillips et al., 2005) as employed in conjunction with the CHARMM27 force field in order to simulate the ensemble of the 255,907 atom system. The initial state was generated from the model by 6,400 steps of conjugate gradient minimization. A 100 ns molecular dynamics simulation was performed to obtain the conformational behaviour of the complete system. The simulations were carried out in the isobaric-isothermal ensemble, maintaining pressure and temperature at 1 atm and 300 K, respectively, using Langevin dynamics (damping parameter of 1 ps^{-1}) and piston approaches. The shake algorithm was used during the simulation. The equations of motion were integrated with a 1 fs time step, using the r-RESPA algorithm electrostatic forces at a slower 2 fs frequency. Long-range interactions were treated using the particle mesh Ewald approach, with an 11 Å cut-off. All molecular dynamics trajectory frames were recorded in interval of 1 ps for a total of 100 ns (i.e. 100,000 frames per trajectory). Once the simulation completed, the conservation of the secondary structure elements during the molecular dynamics simulation was checked using the VMD pluginTimeline.

Pair interaction energies - APEX-GmCSN5, APEX-water and GmCSN5-water interactions during the MD simulation were evaluated by calculating the pair interactions energies with NAMD.

***In situ* APEX labeling of APEX-GmCSN5 protein partners in tobacco leaves** - The BP solution (Iris Biotech GmbH, Germany) was freshly dissolved in DMSO and diluted in an MG buffer (10 mM $MgCl_2$, pH 5.8) to obtain a final concentration of 5 mM. The hydrogen peroxide (H_2O_2, Sigma-Aldrich cat. no. H1009) was freshly diluted with the MG buffer at 1mM before use. Leaves of *N. tabacum* transgenic lines and WT were infiltrated with BP using a blunt-ended 1-mL syringe and incubated for 30 min to allow cell permeability. Leaves were *de novo* infiltrated with H_2O_2 treatment. Negative control samples were similarly prepared by infiltrating solely 10 mM $MgCl_2$ followed by H_2O_2 treatment. Leaves were subsequently detached and frozen in liquid nitrogen. To extract labeled proteins, 15 grams of treated leaf samples were ground manually to a fine powder for 30 min in the cold-room and homogenized with 30 mL of freshly prepared lysis buffer at 4°C [(100 mM Tris, pH 7.7, 150 mM NaCl, 10 mM $MgCl_2$, 0,1% CHAPS, 1 mM EDTA, 1 mM PMSF and 1x Complete protease inhibitors) supplemented with quencher solutions (10 mM sodium azide; 10 mM sodium ascorbate)]. Soluble proteins were extracted upon the addition of quencher reagents to avoid over-biotinylation of distant and unwanted proteins of APEX-GmCSN5 (Rhee et al.,

2013). After centrifugation at 14,000 rpm for 10 min, 500 µl of supernatant samples were added to 30 µl of equilibrated streptavidin magnetic beads (GE Healthcare Life Sciences) and gently rotated for 1 hour at room temperature. Then, extracts were placed on a magnetic rack for 30s to pellet the beads and discard buffer. Beads were washed twice with 1 mL lysis buffer, with quenchers omitted, once with 2M Urea/10mM Tris pH 8.0, and again twice with lysis buffer. Next, the elution step consisted to incubate beads in 30 µL of 3x Laemmli buffer with supplemented 2 mM biotin and 20mM DTT for 10 min and subsequently heat beads at 95°C for 5 min. Twenty millimolar of DTT was added in the samples to ensure a complete disruption of the disulfide bonds. After cooling on ice, samples were briefly spin to pull down the condensation droplets and then beads were pelleted on a magnetic rack to collect eluate fractions. Twelve and six percent of samples were loaded onto 12% SDS-PAGE gel for silver staining to visualize all proteins. To detect biotinylated and APEX-GmCSN5 proteins, remaining eluates were separated by SDS-PAGE gel, transferred to PVDF blotting membrane and blocked with 5% nonfat dry milk in TBST (0,1% Tween-20 in Tris-buffered saline) at 4°C for overnight incubation. The blots were immerged in TBST with antibodies prepared as follows: Streptavidin-HRP 1:1000 (ThermoFisher) and Precision Streptactin-AP 1:5000 (Biorad) for 1 hour at room temperature. Samples were then rinsed four times for 5 min with TBST and once with TBS for 5 min before development. Chromogenic substrates were used for detecting APEX-GmCSN5 using alkaline phosphatase conjugate substrate kit (Biorad) and for biotinylated proteins from the diethyl-amino-benzidine (DAB) solution (Sigma-Aldrich - freshly prepared in DMSO). The identification of proteins by mass spectrometry was performed as described previously (Van Leene et al., 2015). Twenty milliliter of eluates were applied on 12% SDS-PAGE and visualized by coomassie staining. Protein bands were cut into 1 mm slices followed by trypsin digestion and then peptides were analyzed by LC-MS/MS using PLGS database search according to a previous work (Murad et al., 2011).

Protein expression and purification

Escherichia coli BL21 (DE3) Codon Plus (RIL) cells (Novagen) were used to express GST alone and GST-MiPM^{-SP} in 600 ml of LB in 2-L baffle flasks (200 rpm, 37°C). When cell growth reached an OD600 of approximately 1, cells were transferred at 20°C. Protein expression was induced with 0.2 mM Isopropyl β-D-1-thiogalactopyranoside (IPTG) for 16-18 h. Cells were collected by centrifugation (10 min, 4000 rpm, 4°C) and re-suspended in buffer A (50 mM Tris-HCl pH 7,6; 500 mM NaCl) at a volume 30 times lower than the initial culture volume. Afterward, the cells were flash frozen and kept at -80°C until use. The extraction consisted of three sonication steps on ice using a sonifier (Branson Digital) with a 1/8'' tapered microtip (amplitude 20%, time 30'', interval 20''). The protein extract was centrifuged (14000 rpm, 4°C, 10 min), and the supernatant was filtered through a 0.45 µm PVDF membrane (Millipore). The first purification step was performed by HisTrap via Fast Protein Liquid Chromatography (FPLC) (AKTA pure 25 L, GE Healthcare life sciences). The protein extract was loaded onto a HisTrap FF 1 ml (GE Healthcare Life Sciences) column pre-equilibrated with buffer A and 5% buffer B (buffer A + 0.5 M Imidazole). The column was washed until the baseline reached a UV280 absorbance below 5 mMAU. The recombinant protein was eluted by a linear gradient of buffer B (5-100% over 30CV – (column volume)). At this step, two different methodologies were used to purify GST-MiPM^{-SP} or MiPM^{-SP}. For GST-MiPM^{-SP}, the protein was dialyzed (cut-off 14 kDa, 14-16 hr, 4°C) in phosphate buffer saline (PBS pH 7.4 (10 mM Na_2HPO_4, 1.8 mM KH_2PO_4, 2.7 mM KCl, 137 mM NaCl) and loaded onto 1 ml of glutathione sepharose 4B resin (GE Healthcare Life Sciences). The protein was eluted with Tris-buffer pH 8, 50 mM reduced glutathione (GSH) and directly loaded on a GF S75 16/60 gel filtration column equilibrated with PBS buffer. The fractions (E10-E13) corresponding to GST-MiPM^{-SP} were kept at 4°C and used within 2

days. The protein GST alone was similarly purified. For MiPM^{-SP}, the protein was dialyzed (cut-off 3 kDa, 14-16 hr, 4°C) in Prescission buffer (Tris-HCl pH 7, 150 mM NaCl, 1 mM DTT, 0,5 mM EDTA) with the Prescission protease (Merck) at 2U/100 µg of GST-MiPM^{-SP}. The recombinant protein was loaded into a gel filtration column S75 16/60 (GE Healthcare Life Sciences) equilibrated with PBS buffer. The cleaved version of MiPM^{-SP} was kept for 2 days at 4°C until use.

REFERENCES

Branon, T. C., Bosch, J. A., Sanchez, A. D., Udeshi, N. D., Svinkina, T., Carr, S. A., et al. (2017). Directed evolution of TurboID for efficient proximity labeling in living cells and organisms. *bioRxiv*, 196980. doi:10.1101/196980.

Chen, C.-L., Hu, Y., Udeshi, N. D., Lau, T. Y., Wirtz-Peitz, F., He, L., et al. (2015). Proteomic mapping in live *Drosophila* tissues using an engineered ascorbate peroxidase. *Proc. Natl. Acad. Sci.* 112, 12093–12098. doi:10.1073/pnas.1515623112.

Chen, X., Zaro, J. L., and Shen, W.-C. (2013). Fusion protein linkers: property, design and functionality. *Adv. Drug Deliv. Rev.* 65, 1357–1369. doi:10.1016/j.addr.2012.09.039.

Dereeper, A., Guignon, V., Blanc, G., Audic, S., Buffet, S., Chevenet, F., et al. (2008). Phylogeny.fr: robust phylogenetic analysis for the non-specialist. *Nucleic Acids Res.* 36, W465–W469. doi:10.1093/nar/gkn180.

Hung, V., Udeshi, N. D., Lam, S. S., Loh, K. H., Cox, K. J., Pedram, K., et al. (2016). Spatially resolved proteomic mapping in living cells with the engineered peroxidase APEX2. *Nat. Protoc.* 11, 456–475. doi:10.1038/nprot.2016.018.

Hung, V., Zou, P., Rhee, H.-W., Udeshi, N. D., Cracan, V., Svinkina, T., et al. (2014). Proteomic mapping of the human mitochondrial intermembrane space in live cells via ratiometric APEX tagging. *Mol. Cell* 55, 332–341. doi:10.1016/j.molcel.2014.06.003.

Hwang, J., and Espenshade, P. J. (2016). Proximity-dependent biotin labelling in yeast using the engineered ascorbate peroxidase APEX2. *Biochem. J.* 473, 2463–2469. doi:10.1042/BCJ20160106.

Jin, D., Li, B., Deng, X.-W., and Wei, N. (2014). Plant COP9 signalosome subunit 5, CSN5. *Plant Sci.* 224, 54–61. doi:10.1016/j.plantsci.2014.04.001.

Korndörfer, I. P., and Skerra, A. (2002). Improved affinity of engineered streptavidin for the Strep-tag II peptide is due to a fixed open conformation of the lid-like loop at the binding site. *Protein Sci. Publ. Protein Soc.* 11, 883–893. doi:10.1110/ps.4150102.

Lam, S. S., Martell, J. D., Kamer, K. J., Deerinck, T. J., Ellisman, M. H., Mootha, V. K., et al. (2015). Directed evolution of APEX2 for electron microscopy and proximity labeling. *Nat. Methods* 12, 51–54. doi:10.1038/nmeth.3179.

Lin, Q., Zhou, Z., Luo, W., Fang, M., Li, M., and Li, H. (2017). Screening of proximal and interacting proteins in rice protoplasts by proximity-dependent biotinylation. *Front. Plant Sci.* 8. doi:10.3389/fpls.2017.00749.

Lingaraju, G. M., Bunker, R. D., Cavadini, S., Hess, D., Hassiepen, U., Renatus, M., et al. (2014). Crystal structure of the human COP9 signalosome. *Nature* 512, 161–165. doi:10.1038/nature13566.

Lobingier, B. T., Hüttenhain, R., Eichel, K., Miller, K. B., Ting, A. Y., von Zastrow, M., et al. (2017). An Approach to spatiotemporally resolve protein interaction networks in living cells. *Cell* 169, 350-360.e12. doi:10.1016/j.cell.2017.03.022.

Martell, J. D., Deerinck, T. J., Sancak, Y., Poulos, T. L., Mootha, V. K., Sosinsky, G. E., et al. (2012). Engineered ascorbate peroxidase as a genetically encoded reporter for electron microscopy. *Nat. Biotechnol.* 30, 1143–1148. doi:10.1038/nbt.2375.

Murad, A. M., Souza, G. H. M. F., Garcia, J. S., and Rech, E. L. (2011). Detection and expression analysis of recombinant proteins in plant-derived complex mixtures using nanoUPLC-MSE. *J. Sep. Sci.* 34, 2618–2630. doi:10.1002/jssc.201100238.

Phillips, J. C., Braun, R., Wang, W., Gumbart, J., Tajkhorshid, E., Villa, E., et al. (2005). Scalable molecular dynamics with NAMD. *J. Comput. Chem.* 26, 1781–1802. doi:10.1002/jcc.20289.

Qi, Y., and Katagiri, F. (2009). Purification of low-abundance Arabidopsis plasma-membrane protein complexes and identification of candidate components. *Plant J. Cell Mol. Biol.* 57, 932–944. doi:10.1111/j.1365-313X.2008.03736.x.

Reinke, A. W., Balla, K. M., Bennett, E. J., and Troemel, E. R. (2017). Identification of microsporidia host-exposed proteins reveals a repertoire of rapidly evolving proteins. *Nat. Commun.* 8, 14023. doi:10.1038/ncomms14023.

Rhee, H.-W., Zou, P., Udeshi, N. D., Martell, J. D., Mootha, V. K., Carr, S. A., et al. (2013). Proteomic mapping of mitochondria in living cells via spatially restricted enzymatic tagging. *Science* 339, 1328–1331. doi:10.1126/science.1230593.

Roux, K. J., Kim, D. I., Raida, M., and Burke, B. (2012). A promiscuous biotin ligase fusion protein identifies proximal and interacting proteins in mammalian cells. *J. Cell Biol.* 196, 801–810. doi:10.1083/jcb.201112098.

Schmidt, T. G. M., and Skerra, A. (2007). The Strep-tag system for one-step purification and high-affinity detection or capturing of proteins. *Nat. Protoc.* 2, 1528–1535. doi:10.1038/nprot.2007.209.

Sharp, K. H., Mewies, M., Moody, P. C. E., and Raven, E. L. (2003). Crystal structure of the ascorbate peroxidase-ascorbate complex. *Nat. Struct. Biol.* 10, 303–307. doi:10.1038/nsb913.

Van Leene, J., Eeckhout, D., Cannoot, B., De Winne, N., Persiau, G., Van De Slijke, E., et al. (2015). An improved toolbox to unravel the plant cellular machinery by tandem affinity purification of Arabidopsis protein complexes. *Nat. Protoc.* 10, 169–187. doi:10.1038/nprot.2014.199.

Webb, B., and Sali, A. (2017). Protein structure modeling with MODELLER. *Methods Mol. Biol. Clifton NJ* 1654, 39–54. doi:10.1007/978-1-4939-7231-9_4.
